# The polarization of literary censorship in the U.S

**DOI:** 10.1371/journal.pone.0332240

**Published:** 2025-09-23

**Authors:** Adam Szetela, Shiyu Ji, Michael Walton Macy

**Affiliations:** 1 Department of Literatures in English, Cornell University, Ithaca, New York, United States of America; 2 Department of Sociology, Cornell University, Ithaca, New York, United States of America; 3 Departments of Sociology and Information Science, Cornell University, Ithaca, New York, United States of America; Xi'an University of Posts and Telecommunications, CHINA

## Abstract

Literary censorship is not new, but the recent resurgence in the U.S. differs from earlier post-war controversies, many of which were bipartisan and focused on protecting children from exposure to sexual and graphic material. Current censorship controversies in the U.S. appear to be much more partisan and ideological, focused on protecting children from politically offensive ideas. Anecdotally, the political right is portrayed as attacking literature in the name of conservative values like preserving the traditional family from alternative expressions of sexuality and sexual preference, with the left targeting material regarded as contrary to progressive values like tolerance of diverse cultural identities. However, these ideological rationales largely reflect the rhetoric of political activists and have not been empirically tested among the broader public. We conducted two studies designed to measure the polarization of support for literary censorship among the voting-age population of the U.S. Surprisingly, both studies cast doubt on the ideological divisions apparent in activist rhetoric. The survey findings from Study 1 indicate widespread opposition to literary censorship that spans ideological divisions, but both liberals and conservatives were more inclined to support censorship of materials that deviated from their respective values and beliefs. The experiment in Study 2 revealed differences in participants’ responses to liberal and conservative criticisms but little difference in the attitudes of liberal and conservative participants. However, liberals were marginally more likely than conservatives to agree with ideologically aligned literary criticism.

## Introduction

In his coda to *Fahrenheit 451*, Ray Bradbury wrote: “There is more than one way to burn a book. And the world is full of people running about with lit matches” [1] (p. 209). Today, in the U.S., the fires often start on social media, with progressives accusing literature of racism, sexism, homophobia, antisemitism, and Islamophobia, and conservatives accusing literature of being anti-Christian, anti-*American*, man-hating, and anti-family. Fueled by online vitriol, the firestorms then spread from talk to action, with books being pulled from public libraries and public schools, from brick-and-mortar bookstores, as well as Amazon and eBay [2]. Faced with public protests, publishers have cancelled literature they had previously agreed to publish and even retracted previously published literature. As *The New York Times* editorial board put it in 2022, “America has a free speech problem.” [3]

These cancellations, retractions, and rewrites show no sign of abating. For example, the American Library Association’s Office of Intellectual Freedom “documented 1,269 demands to censor library books and resources in 2022, the highest number of attempted book bans since ALA began compiling data about censorship in libraries more than 20 years ago.” [4] Their preliminary data for 2023 illustrates a “record surge of challenges in public libraries.” [5]

These campaigns extend beyond dissemination of objectionable material to target the source. For example, PEN America’s 2023 report on “booklash” concluded, “Some readers, writers, and critics are pushing to draw new lines around what types of books, tropes, and narrative conventions should be seen as permissible... Authors and publishers have felt compelled to respond to this intensifying form of literary criticism, which is amplified through online discourse.” [2]

The change is not only in the number of incidents but in the justifications. Historically, both the right and the left have targeted sexual content, criticized not only as immoral but also as dangerous to children’s mental health, leading to calls like “Ban this Filth That Poison Our Children” (pp. 9–11) [6]. In the 1950s, comic books like *Tales from the Crypt* came under fire as a genuine public health concern among Americans on the right and the left (p.11) [6]. In response, the U.S. Senate held a bipartisan hearing in 1954 on comic books. The “Approved by the Comics Code Authority” label on the front of comic books is a legacy of that campaign, justified by the need to protect children from exposure to psychologically harmful content for which they are not developmentally prepared. As media scholar Henry Jenkins put it, there has been a tendency to see “children only as potential victims of the adult world or as beneficiaries of paternalistic protection” [7] (quoted, p. 7). In *The Seduction of the Innocent*, psychologist Fredric Wertham, who testified in the 1954 hearings, noted that “all this would be funny if the happiness and mental development of children were not involved” [8] (p. 38).

This mid-century bipartisan consensus in support of censorship reflected the relative absence of ideological polarization between liberals and conservatives. In the immediate postwar years, liberals in the U.S. supported federal government intervention to reduce unemployment, poverty, and racial segregation, while conservatives believed these policies were responsibilities of state and local government and that a free market was key to prosperity. Nevertheless, these ideological divisions cut across party identities and alignments, allowing liberals and conservatives to find bipartisan common ground in support of Cold War containment of a widely perceived threat of communist influence at home and abroad, while neither party was united in opposition to *de fact*o or *de jure* racial segregation [9,10]. Even in the tumult of the late 1960s, activists on the left targeted both Democrats and Republicans, with only a moderate increase in partisan polarization between 1972 and 2004 [11]. (NB: We use the terms “ideological polarization” and “partisan polarization” to refer to divisions of social opinions between self-identified liberals and conservatives [12,13] and between Democrats and Republicans [14], respectively.)

By the 2020s, however, opinion surveys revealed a significant widening of the ideological divide [15], with partisan identities becoming more closely aligned with ideological positions [16], and with heightened affective polarization, characterized by increased partisan hostility [15,17,18].

These divisions are evident as well in the language used by activists to criticize children’s literature. On the left, the main targets are books that progressives believe are reinforcing racism, sexism, and homophobic intolerance [19]. (NB: We use “liberal” to refer to center-left support for an expanded pragmatic role of the federal government and “progressive” to refer to those further to the left who also focus on empowerment of disadvantaged groups based on race, gender, and sexual identity. We use “conservative” and “right wing” analogously, to refer to center-right and extreme-right, respectively.) For example, in a book recently honored by the Children’s Literature Association and the Association of American Publishers, *Was the Cat in the Hat Black? The Hidden Racism of Children’s Literature, and the Need for Diverse Books*, distinguished literary critic Philip Nel called out the “hidden racism of children’s literature,” arguing that books can “infect developing young minds” [20] (p. 63). “If the man who killed Trayvon Martin had read *The Snowy Day* as a kid,” writes Christopher Myers, a finalist for the National Book Award who launched the Make Me a World imprint at Penguin Random House, “would it have been as easy for him to see a seventeen-year-old in a hoodie, pockets full of rainbow candies and sweet tea, as a threat?” [21].

On the right, sexuality continues to be an issue, but the targeting is now focused more narrowly on literature that violates the normative model of cisgendered heterosexuality. For example, Tennessee HB 800, a bill introduced by Republican house representative Bruce Griffey, proposed to ban books that “promote, normalize, support, or address lesbian, gay, bisexual, or transgender (LGBT) issues or lifestyles” [22]. The right has also targeted Critical Race Theory, a term taken out of its legal context and now used to vilify books and educational materials that promote diversity. As PEN America notes in its recent report on educational gag orders, “In state after state, primary and secondary teachers and pre-service teacher educators have strongly attested that, as an intellectual framework, Critical Race Theory is not taught in elementary, middle or high schools, insisting that critics have conflated the academic theory taught in colleges (and law schools in particular) with other diversity initiatives” [23].

Of the 695 censorship attempts and 1,915 challenges in the American Library Association’s 2023 preliminary report [24], the “vast majority of challenges were to books written by or about a person of color or a member of the LGBTQIA+ community” [25]. When conservatives targeted Alex Gino’s *George*, it was because the book did not reflect “the values of our community” [26]. When progressives attacked *Blood Heir*, it was due to allegations of racial insensitivity, a transgression of core values in the progressive community [27].

In short, literary censorship has expanded far beyond the need to protect children from exposure to graphic violence and sexuality to target “an increasingly diverse array of opinions and attitudes,” reflecting the “polarization occurring via social, cultural, and political alignments” (p. 507) [28]. This alignment of previously cross-cutting policy positions with symbolic expressions of cultural identity led Steven Pinker to ask “Why on earth should people’s beliefs about sex predict their beliefs about the size of the military? What does religion have to do with taxes? Whence the linkage between strict construction of the Constitution and disdain for shocking art?” (p. 154) [29]. Alarmingly, the battleground of this “culture war” is spreading beyond schools and libraries to the publishing industry itself.

Our research is motivated by the concern that polarization has come to encompass attitudes toward literature and its censorship. Censorship in the 1950s involved a bipartisan coalition of concerned literary critics, teachers, and the broader public. In today’s polarized political landscape, censorship “happens whenever some people succeed in imposing their political or moral values on others by suppressing words, images, or ideas that they find offensive,” as noted by the non-partisan National Coalition Against Censorship [30]. The current wave of literary censorship in the U.S. appears to be driven not by a bipartisan concern over the purported psychological harm from exposure to sexual content, as happened in the 1950s, but by extreme ideological polarization.

We conducted two studies designed to test the hypothesized polarization of literary censorship in the broader U.S. population. The first study used a survey to gauge attitudes about the censorship of children’s literature, exploring the influence of ideological and demographic factors. The second study used endorsement of ideological literary criticisms (e.g., “misogynistic” vs. “man-hating”) to drill down into the underlying objections that might motivate the willingness to censor, outside the context of protecting children from inappropriate content and without the confounding effects of First Amendment sensitivities. Both studies add to a growing body of academic research that attempts to discern the mechanisms that fuel literary censorship [19,31–33].

## Study 1: Public support for censorship of children’s literature

### Methods

Study 1 explored attitudes regarding the censorship of children’s literature through a survey conducted on the Prolific crowdsourcing platform, with English-speaking U.S. participants aged 18 years or older. Although we obtained a demographically representative sample, the participants were not recruited from the general population and the sample was disproportionately liberal (which we corrected by reweighting the data). The study was exempted from review by Cornell University’s Institutional Review Board (Protocol ID: 2112010750). Electronic consent was obtained after participants were shown a digital consent form.

In contrast to the bipartisan support for censorship in an earlier era, literature has increasingly become the focus of ideological controversy [2,34,35]. On the left, critics have criticized K-12 schools for assigning books that valorize the founding fathers (white men who owned slaves) and a “curriculum of dead white men” [36]. They have criticized white and heterosexual authors for writing from the perspective of queer characters and characters of color [37]. They have charged publishers with giving an unfair advantage to white authors in the acquisitions process and pressured publishers to retract, rewrite, and discontinue books regarded as recycling racist, sexist, and homophobic tropes [38,39].

On the right, activists have pressured local and state officials to remove books rom K-12 schools they regard as harmful to young people, including books with LGBTQ+ characters and books about racism, calling instead for more children’s books with Christian themes [5,40]. Both sides criticize each other for advocating censorship, but conservatives in particular charge the left with creating a “cancel culture” and claim to be more tolerant of children’s books they dislike [41,42].

For Study 1, we condensed these ideologically aligned controversies into 15 statements, including statements that measure attitudes about censorship more generally and willingness to censor books that might upset some parents. Participants responded to these statements on a five-point Likert scale ranging from –2 (“strongly disagree”) to +2 (“strongly agree”). Items 1–5 were worded so that liberals might be more likely to agree. Items 6–10 were worded so that conservatives might be more likely to agree. Items 11–15 were worded so that agreement might be equally likely, regardless of the participant’s ideological alignment. The classification of statements by ideological alignment was based on the ideological identity of the sources of the observed criticisms. We did not pre-test the items to confirm their ideological alignment with the expected ideological identities of participants because that alignment is what the study is intended to test. Items 8, 11, and 13 were worded so that agreement would indicate concerns about censorship or support for tolerance of disliked books, or both. Item 10 measures participants’ beliefs about differences between liberals and conservatives in support for censorship. For the remaining items, agreement indicates support for censorship. Each participant was presented with the 15 items in random order. The list of survey items is shown in Table 1.

**Table 1:** List of survey items based on recent controversies over censorship of children’s literature, classified by the association with ideologically self-identified groups [2,34,35]. The items were randomly ordered in the survey but are numbered here for clarity in the presentation of results.

Ideological Leaning		
Liberal	Conservative	Independent/Other
1. Public elementary schools should not assign a history book about the important contributions of famous white men who owned slaves.	6. Public elementary schools should not assign a book about a transgender character who inspires children to celebrate all gender identities.	11. Censorship of children’s books is a serious problem in the U.S..
2. Publishers should discontinue a children’s book accused of racism, sexism, or homophobia.	7. LGBTQ+ books are harmful to children.	12. It is OK for adults to burn children’s books as a public protest against books they dislike.
3. Publishing companies should reject a children’s novel written by a heterosexual white man who writes from the perspective of a queer woman of color.	8. Public elementary schools should assign more children’s books about Christian themes.	13. Adults should not criticize a children’s book they haven’t read.
4. K-12 public schools are assigning too many books written by white male authors.	9. K-12 public schools are assigning too many books about racism.	14. A public library should remove a children’s book if they think it might upset some parents.
5. Publishers are more likely to accept a children’s book if the author is a white male.	10. Compared to liberals, conservatives are more tolerant of children’s books they dislike.	15. Public elementary schools should not assign a book about someone who commits suicide.

Following the survey, participants were asked about their demographic background, including political ideology, parenting status (whether they had children under 18), age, gender, race, religious affiliation, income bracket, educational attainment (including whether they were currently in college or university), and sexual orientation. Details about the data collection and survey procedure are available in S1 File. Demographic questions are listed in S2 File. The demographics of the participants are reported in S1 Table.

Out of the 891 initial participants, 864 completed the survey (a 97% completion rate), of whom 32 participants failed to indicate their ideology and were dropped from the analysis. The sample was weighted to match the ideological distribution of the adult U.S. population as measured by the General Social Survey [38].

The results provide a descriptive account of differences between liberals and conservatives in support for censorship, along with a test of a demographic explanation. We begin by reporting the bivariate results, followed by the results of a multivariate model that measures ideological differences after taking into account demographic differences.

## Study 1 Results

Table 2 reports support for censorship of children’s books across all participants, as well as separately for liberals, conservatives, and moderates. We conducted two-tailed one-sample t-tests for each ideological group. The results show overall opposition to censorship among all participants, regardless of the participant’s self-identified ideology. Participants supported censorship only for children’s books about someone who commits suicide (0.267, *p* < 0.001). The strongest opposition to censorship is for item 13, about a book the critics have not read (1.134, *p* < 0.001), followed by item 1 about a book that highlights contributions of white slave owners (−0.906, *p* < 0.001), item 14 about a book that might upset parents (−0.878, **p* *< −0.001), and item 12 about book-burning (−0.868, *p* < 0.001). As a group, participants also agreed that censorship of children’s books is a serious problem (0.384, *p* < 0.001).

**Table 2:** Mean survey response by ideology.

Item	All^1^	Liberal	Moderate	Conservative
1. Public elementary schools should not assign a history book about the important contributions of famous white men who owned slaves.	−0.906^***^	−0.571^***^	−0.908^***^	−1.196^***^
2. Publishers should discontinue a children’s book accused of racism, sexism, or homophobia.	−0.022	0.085	−0.031	−0.087
3. Publishing companies should reject a children’s novel written by a heterosexual white man who writes from the perspective of a queer woman of color.	−0.303^***^	−0.394^***^	−0.276^***^	−0.027
4. K-12 public schools are assigning too many books written by white male authors.	−0.495^***^	0.004	−0.442^***^	−0.967^***^
5. Publishers are more likely to accept a children’s book if the author is a white male.	−0.225^***^	0.221^***^	−0.276^***^	−0.549^***^
6. Public elementary schools should not assign a book about a transgender character who inspires children to celebrate all gender identities.	0.110^*^	−1.165^***^	0.252^*^	1.071^***^
7. LGBTQ+ books are harmful to children.	−0.231^***^	−1.499^***^	−0.147	0.826^***^
8. Public elementary schools should assign more children’s books about Christian themes.	−0.508^***^	−1.278^***^	−0.509^***^	0.163
9. K-12 public schools are assigning too many books about racism.	−0.341^***^	−1.111^***^	−0.307^***^	0.299^***^
10. Compared to liberals, conservatives are more tolerant of children’s books they dislike.	−0.782^***^	−1.581^***^	−0.699^***^	−0.147
11. Censorship of children’s books is a serious problem in the U.S..	0.384^***^	1.136^***^	0.276^**^	−0.147
12. It is OK for adults to burn children’s books as a public protest against books they dislike.	−0.868^***^	−1.301^***^	−0.798^***^	−0.554^***^
13. Adults should not criticize a children’s book they haven’t read.	1.134^***^	1.520^***^	1.184^***^	0.712^***^
14. A public library should remove a children’s book if they think it might upset some parents.	−0.878^***^	−1.421^***^	−0.877^***^	−0.380^***^
15. Public elementary schools should not assign a book about someone who commits suicide.	0.267^***^	−0.184^***^	0.362^***^	0.538^***^

* *p* < 0.05** *p* < 0.01, *** *p* < 0.001

One sample two-tailed *t-*test was conducted for each item and each ideology group.

^1^The full sample was reweighted to match the distribution of liberals and conservatives in the 2022 General Social Survey [43]. The other three data columns remained unweighted.

The aggregated responses closely resemble those of self-identified moderate participants, who fall midway between the responses of liberals and conservatives on every item. However, aggregated responses obscure consistent differences between liberal and conservative participants that tend to cancel each other out. We conducted two-tailed two-sample t-tests to compare the mean differences in responses between liberals and conservatives. All reported between-group differences are statistically significant, with *p*-values less than 0.001. On items 6 and 7, conservatives strongly support censorship of books featuring an inspiring transgender character (1.071, *p* < 0.001) or books with LGBTQ+ content (0.826, *p* < 0.001), while liberals even more strongly disagree (−1.165 and −1.499 respectively, *p* < 0.001). On item 9, conservatives modestly support the censorship of books about racism (0.300, *p* = 0.001), while liberals strongly disagree (−1.111, *p* < 0.001). On item 5, liberals believe too many books are published by white male authors (0.221, *p* < 0.001), while conservatives disagree (−0.549, *p* < 0.001). On item 8, liberals strongly oppose the publication of more books with Christian themes (−1.278, *p* < 0.001), while conservatives do not have a strong opinion (0.163, *p* = 0.073). Not surprisingly, liberals strongly disagree that conservatives are more tolerant (−1.581, *p* < 0.001), while conservative responses were not statistically significant (−0.147, *p* = 0.078). Responses to item 11 indicate that, compared with conservatives, liberals (1.136, *p* < 0.001) tend to be more concerned with children’s book censorship (−0.147, *p* = 0.127). However, both liberals and conservatives support the censorship of some children’s books – just not the same ones.

These results demonstrate consistent differences between liberal and conservative participants about which books to censor, but the explanation is not necessarily grounded in ideology. What appear to be ideological differences might instead reflect the effects of demographic covariates – notably race, gender, age, education, income, parenthood, sexual orientation, and religion – which can influence participants’ views and experiences in ways that shape their responses to censorship.

We tested the demographic explanation using a multivariate logistic regression model. Table 3 reports ideological and demographic differences in support for censorship, along with ideological differences after controlling for demographic covariates. The table includes two measures of association: 1) the Spearman rank-order correlation between an ordinal measure of ideology (ranging from extremely liberal to extremely conservative) and the strength of agreement (ranging from strongly disagree to strongly agree), and 2) the odds that a conservative participant would agree with an item compared to a liberal (using binary measures of ideology and agreement).

**Table 3:** Spearman rank correlation coefficients between (1) Likert-scale responses to survey items and participant attributes, and (2) odds ratios from a binary logistic regression. For the odds ratio (OR), we collapsed the Likert scale by grouping together “Strongly Agree” and “Agree” and grouping together “Strongly Disagree,” “Disagree,” and “Neither Agree nor Disagree”. Ideology uses a binary measure of Liberals or Conservatives, *N* = 669. We controlled for demographic traits using the normalization procedure recommended by Gelman [44]. In the logistic regression, education was imputed as the number of years needed to complete the degree corresponding to the qualitative response, and income was imputed as the mean income within the income bracket.

		Binary Logistic Regression OR^+^													
	Correlation With Conservative Identity	Ideology (Ref. Liberal)		Gender (Ref. Female and Others)	Race (Ref. White)		Religion (Ref. No Religion)							Sexual Orientation (Ref. Heterosexsual)	
Item	Rho	Conservative	Age	Male	Black	Other		Catholic	Protestant	Other	College	Parent	Education	All Other	Income
1	−0.238^***^	0.353^***^	0.633	0.781	2.504^**^	3.214^***^		1.819	1.912^*^	1.716	1.254	1.806^*^	0.944	2.298^**^	1.031
2	−0.060	0.737	0.673^*^	0.814	1.589	1.261		2.015^*^	1.200	0.935	1.851^*^	1.226	0.759	0.905	1.242
3	0.100^**^	1.774^*^	0.639^*^	0.695	2.337^**^	0.764		0.943	2.552^***^	2.182^**^	1.086	1.067	0.755	1.179	1.240
4	−0.412^***^	0.188^***^	0.786	0.632^*^	1.636	1.588		0.64	0.558	1.534	1.235	1.045	1.807^*^	2.607^***^	0.829
5	−0.337^***^	0.253^***^	0.576^**^	0.807	2.011^**^	1.225		1.227	0.998	1.319	0.834	1.240	0.996	1.476	0.712
6	0.659^***^	−23.654^***^	1.260	1.074	5.043^***^	1.731		1.937	2.921^***^	2.261^*^	1.017	1.025	0.951	0.542	1.136
7	0.701^***^	38.954^***^	1.327	1.501	10.337^***^	1.356		1.984	4.768^***^	3.868^***^	1.314	1.219	0.557	0.336^*^	1.179
8	0.535^***^	9.407^***^	2.278^**^	1.017	9.833^***^	1.009		11.074^***^	9.964^***^	5.094^**^	0.514	1.556	0.427^**^	1.003	1.875^*^
9	0.547^***^	11.12^***^	1.726	1.950^*^	0.863	2.140		2.806^*^	2.370^*^	1.444	1.737	1.187	0.804	1.007	0.679
10	0.570^***^	16.746^***^	1.507	1.675	1.870	0.882		2.403	1.143	0.861	2.847^**^	2.847^***^	1.584	1.814	0.506
11	−0.485^***^	0.152^***^	1.878^**^	0.674^*^	0.443^**^	0.732		0.723	0.507^**^	0.905	1.266	1.266	1.134	1.276	1.01
12	0.284^***^	4.644^***^	0.861	2.607^***^	1.038	0.972		0.495	0.885	0.358^*^	1.247	1.247	1.486	1.533	0.811
13	−0.361^***^	0.175^***^	1.569	0.537^*^	0.593	1.698		0.799	0.502^*^	0.775	1.174	1.174	0.976	0.928	0.725
14	0.442^***^	4.087^***^	0.960	1.650	5.865^***^	3.400^**^		2.482	3.117^*^	1.428	1.934	1.934	0.484^*^	0.219	0.909
15	0.273^***^	2.353^***^	1.028	0.885	2.242^**^	1.382		1.962^*^	2.013^**^	1.288	1.419	1.419	0.736	1.2	0.835

*p < 0.05** *p* < 0.01, *** *p* < 0.001

^+^All discrete numerical and continuous variables are normalized by two standard deviations, following [44]

We also measured the odds that a participant with a given demographic attribute would agree with the item. Following Gelman [44], we obtained normalized odds ratio estimates for the three non-binary demographic variables (age, education, and income). Normalized odds ratios allow us to compare the likelihood of an event occurring across different groups, adjusting for demographic differences.

The results were in the expected ideological direction. Controlling for demographic differences, on items 6 and 7 (Table 3), conservatives were over 20 times more likely than liberals to oppose the assignment of books about transgender characters (OR = 23.654, ρ = 0.659, *p* < 0.001), and over 30 times more likely to agree that LGBTQ+ books are harmful to children (OR = 38.934, rho = 0.701, *p* < 0.001). Liberals were more willing to reject history books highlighting significant contributions of white slave owners (item 1; OR= 0.353, ρ = – 0.238, *p* < 0.001, Table 3), while conservatives were more likely to agree that K-12 schools assign too many books about racism (item 9; OR=11.120, ρ = 0.547, *p* < 0.001, Table 3). Liberals were more likely to oppose school assignment (item 4) and publication (item 5) of books authored by white males (OR = 0.188 and.253, ρ = – 0.412 and – 0.337, both *p* < 0.001, Table 3), and more likely to oppose publication of more books with Christian themes (item 8, OR = 9.407, ρ = 0.535, *p* < 0.001, Table 3).

This ideologically consistent pattern suggests that liberals and conservatives are surprisingly similar in their views about censorship: both sides are willing to let children read books that reflect their own values but not those that might expose children to ideological contamination. Moreover, item 3 is the exception that proves the rule: Conservative and liberal participants were about equally inclined to reject a book focusing on a queer woman of color authored by a heterosexual white man, a book that might offend both sides but for very different reasons. Liberals may have found the book problematic due to concerns about the author’s ability to authentically represent the experiences of a queer woman of color, reflecting the “politics of representation” as a concern on the left [45], whereas conservatives may have been opposed to the book’s focus on LGBTQ+ themes, given the widespread attacks on LGBTQ+ books and books about POC from the right. As the American Library Association notes in its 2023 report, “titles representing the voices and lived experiences of LGBTQIA+ and BIPOC individuals made up 47% of those targeted in censorship attempts” [5]. Simply put, liberals may have reacted out of concerns about authenticity, while conservatives may have reacted out of concerns about a queer woman of color.

The five ideologically neutral items provide a measure of liberal and conservative support for censorship that is not ideologically motivated. Conservative participants were more willing than were liberals to favor censorship on all five ideologically neutral items (i.e., more likely to agree on items 12, 14, and 15 and less likely to oppose censorship on items 11 and 13). For example, while liberal as well as conservative participants agree that children’s book censorship is a serious problem in the U.S. (item 11), conservatives were far less likely to agree (OR = 0.152, *p* < 0.001) and the rank-order correlation between ideology and agreement was negatively signed (ρ = – 0.485, *p* < 0.001). Similarly, both sides agreed that adults should not criticize a book before reading it (item 13), but conservatives were significantly less likely to agree (OR = 0.175*,* ρ = – 0.361, *p* < 0.001).

The other three ideologically neutral items more directly measured willingness to participate in censorship activities: book burning (item 12), removing a book from a public library in response to parental complaints (item 14), and not including a book in the school curriculum that mentions suicide (item 15). Conservatives were more likely to agree with burning a children’s book as a public protest (OR = 4.644, ρ = .284, *p* < 0.001), removing a book from a public library (OR= 4.087, ρ = 0.442, *p* < 0.001) and the school curriculum (OR = 2.353, ρ = 0.273, *p* < 0.001). Ironically, while conservatives are generally more censorious, responses to item 10 indicate that they believe they are more tolerant of children’s books they might personally find objectionable (OR = 16.746, ρ = 0.57, *p* < 0.001).

Table 3 also reports demographic differences in support for censorship over and above the differences attributable to ideological beliefs. An important finding is that ideology is statistically associated with more beliefs about censorship (14 out of 15 items) than any of the demographic attributes (age: 5 of 15; gender: 5; race: 9; religion: 12; education: 3; parenthood: 2; sexual orientation: 2; and income: 1).

In conclusion, Study 1 measured differences between self-identified liberal and conservative participants in their willingness to censor children’s books, depending on ideologically aligned attributions about the content of the book or its authors. We chose children’s books because of the history of bipartisan support for protecting children from graphic content that might be harmful to their mental health. The results provide limited support for the hypothesis that censorship has become ideologically polarized, based on the need to protect children from exposure to books perceived as threatening to ideologically aligned core values. The survey indicated widespread opposition to literary censorship that spans ideological divisions, but both liberals and conservatives were more inclined to support censorship of materials that deviated from their respective values and beliefs.

## Study 2: Liberal and conservative responses to ideological literary criticisms

Study 2 extends our investigation of the polarization of censorship using a controlled experiment instead of a survey and using agreement with literary criticisms of poetry to measure potential support for censorship outside the context of protecting children from inappropriate content and without the confounding effects of First Amendment sensitivities. More precisely, our experiment makes it possible to isolate participants’ responses to criticisms that differ on one of eight ideologically aligned core values: that the poem was racist, misogynistic, antisemitic, homophobic, anti-family, anti-Christian, anti-American, or man-hating.

### Methods

The experiment was conducted via the Prolific crowdsourcing platform between March 7, 2023, and March 17, 2023, among English-speaking users living in the U.S. aged 18 years or older. (Participant demographics are reported in S2 Table in supplemental materials). The study received exemption from Cornell University’s Institutional Review Board (Protocol ID: 2112010750), and electronic consent was obtained after participants were shown a digital consent form. Among 893 participants who signed up for the study, 815 completed the experiment, for a completion rate of 91.3%. Each participant was paid about $1.89 USD.

#### Poem selection and presentation.

During the experiment, all participants were asked to evaluate four short poems in random order. Pseudonyms were used for the poets to prevent participants from being influenced by name recognition or biases associated with a specific poet, which could affect their perceptions of the criticism. None of the four poems contained salient ideological content, as previously pre-tested with a different set of participants. However, three of the poems had been accused of moral transgressions in literary publications: “Sweeney among the Nightingales” by T. S. Eliot had been accused of antisemitism; “Avenues” by Eugen Gomringer had been accused of sexism; and “How-To” by Anders Carlson-Wee had been accused of racism. The experiment also included a poem that was not accused of moral transgressions in any literary publication (“A Supermarket in California” by Allen Ginsberg). Since this poem was substantially longer than the others, we used an excerpt so that its length was similar to the other three poems. (The full poems, including the excerpt from “Supermarket,” are included in S3 File.)

#### Treatment conditions.

Participants were randomly assigned to one of two treatment conditions in a between-participant design. In the exposure condition (*N* = 635), for each poem, participants read the poem followed by a literary criticism. In the no-criticism condition (*N* = 180), participants were randomly assigned to evaluate the poem based solely on the poem itself, without exposure to literary criticism. The no-criticism condition provided a baseline needed to rule out the possibility that participant responses to the poem might be similar to those of the literary critic, even without being exposed to the views of the critic. The differences in responses between the two conditions can then be attributed solely to the effects of exposure to literary criticism.

Each poem could be associated with four possible criticisms, one positive and three negative. The positive criticism was not ideologically aligned and was included as a benchmark to compare differences between liberal and conservative responses when no ideological content was present. Positive criticisms used terms such as “virtuous, wholesome, enlightened, and noble,” which appeal equally to liberals and conservatives. Two of the three negative criticisms were ideological, one left-wing and one right-wing, with the third unaligned, focused on technical aspects of the poem. The technical criticism served as an additional benchmark for measuring responses to ideological criticism. Left-wing criticisms included accusations of racial, misogynistic, antisemitic, or homophobic content, while right-wing criticisms accused the poem of being anti-family, anti-Christian, anti-American, or man-hating. The ideological alignment of each criticism was validated using a pre-test. In the pre-test, 53 participants, separately recruited from Prolific, rated the political leaning of each ideological criticism—presented without its corresponding poem—on a 7-point scale ranging from “Extremely Conservative” to “Extremely Liberal.” The results confirmed that participants’ perceptions of each criticism’s ideological alignment matched the intended design (detailed procedures and results are provided in S5 File).

**Fig 1:**
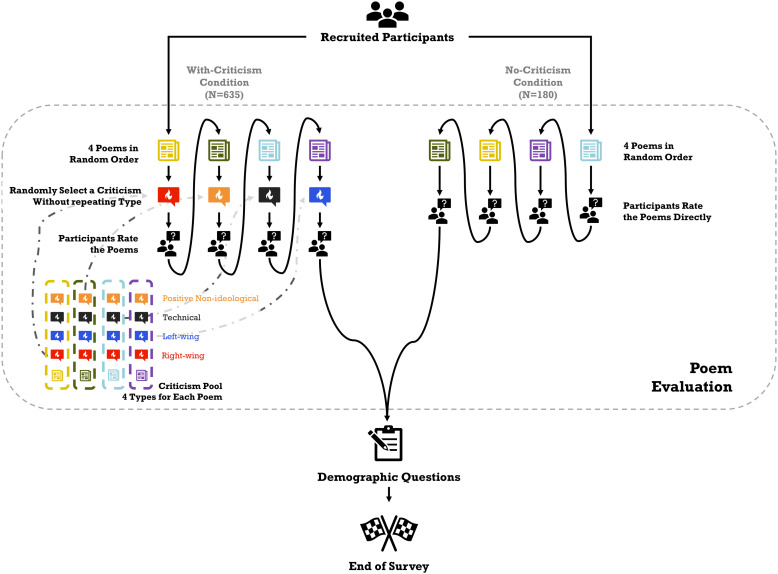
Treatment design of the experiment. Participants were assigned to either the criticism condition (*N* = 635) or the no-criticism condition (*N* = 180). In both conditions, each participant read four poems in random order. In the criticism condition, each poem was accompanied by one of four criticisms: positive and negative non-ideological criticisms, and liberal and conservative negative criticisms. Each type of criticism occurred exactly once per participant to ensure balanced exposure. Attention checks were used to ensure participant engagement.

Each participant was exposed one time to each of the four types of criticism, in a randomized sequence, as a within-participant manipulation. The design of the between-participant and within-participant treatment conditions is summarized in Fig 1.

After reading each poem and criticism (if any), participants were asked to complete attention checks to ensure they had read the materials carefully. The attention check consisted of simple questions about the content of the poem and/or criticism (if a criticism was presented). (A full list of the attention check questions is provided in S3 File.) Participants were warned that repeated mistakes on the attention checks would lead to dismissal from the study without compensation. Those who passed the attention check were then presented with four questions about the poem. This same sequence—poem, criticism (if any), attention check, and four questions about the poem—was repeated for each of the poems four times.

#### Dependent Measure: Agreement with the criticism.

Directly asking participants about their agreement with a randomly assigned criticism could introduce experimenter-demand bias, where participants may feel pressured to conform to perceived expectations. To mitigate this, we designed a word-choice question to implicitly gauge participant agreement. Not directly asking for agreement also allows us to evaluate the level of agreement in the absence of criticism, providing a more nuanced understanding of participants’ natural responses.

To estimate the level of agreement with each type of criticism, participants were asked to select one word from a list of eight randomly ordered words (plus “None of the above”) that best described their assessment of the poem. These words were chosen to represent a balanced range of responses, including the word associated with the criticism, with an equal number of positive and negative choices.

##### Word list construction:

Each poem could be linked to four different types of criticisms, and each criticism was associated with a specific word from a list of eight words. The word list included all four words related to each of the four criticisms, regardless of whether participants were exposed to that specific criticism. This ensured consistency across participants and avoided bias that might be introduced by having systematically different lists for different criticisms.

To complete the list, four additional words were randomly selected from a common pool to maintain an even balance between positive and negative descriptors. Four of the words were technical while the other four were ideologically aligned, to present participants with a balanced set of choices. (Details about the word list construction are elaborated in S4 File.)

To illustrate how the word list was constructed, consider the following example. Suppose a participant reads a poem that is randomly paired with the criticism that the poem is “antisemitic” (a criticism validated in the pre-test as liberal-aligned). For other participants, this same poem might also be paired with three other criticisms: “anti-American” (conservative), “enlightened” (positive), and “poorly written” (technical). Thus, regardless of which criticism is presented to the participants, all participants will see these same four words in the eight-word list (along with four words that are not used in any of the criticisms). The latter are used to balance the word list with four positive and four negative words, in order to avoid bias that could make participants more critical or less critical, depending on the imbalance in the number of positive and negative words. We balanced the list of one positive (technical) and three negative words (left, right, and unaligned) by randomly drawing three positive words and one negative word from a pool of non-ideological words (listed in S4 File).

##### Responses in the no-criticism condition as a base-line measure:

Participant agreement with each four types of criticism could be influenced by the content of the poem, such that participants might hold the same viewpoint as the criticism even without having read it. We measured the baseline response as the probability in the no-criticism condition of choosing the word associated with a given criticism. For each of the four types of criticism, the effect was calculated as the difference between the proportion selecting the word associated with that criticism and the corresponding proportion in the no-criticism condition. We used a bootstrap procedure with 300,000 replications to estimate the mean treatment effect for each type of criticism. More details about the bootstrap procedure are available in S6 File.

#### Other response measures.

Given that most people are unfamiliar with literary criticism, we expected participants to agree with the criticism through deference to a perceived literary expert. This deference allows for a conservative test of the effects of ideological alignment. For a less conservative test, we also included three response measures that did not require literary expertise: whether participants found the poem easy to understand, whether they liked the poem, and whether they agreed with the editor’s decision to publish it. These responses were measured on a seven-point Likert scale ranging from −3 to +3, with higher values indicating a more positive response.

After completing their evaluations of all four poems, participants were asked to indicate their ideological position using the same seven-point scale as in Study 1. Using these scores, we classified participants into three groups: liberals, conservatives, and moderates. We also collected demographic information consistent with Study 1, including age, gender, income level, religion, current college/university attendance, and sexual orientation (see S1 File for item wordings).

## Study 2 Results

### Ideological differences in the No-criticism condition

Table 4 reports the baseline assessment of the poem as measured by agreement with the words associated with the four types of criticism, in the absence of exposure to the criticism (i.e., in the no-criticism condition). Table 5 reports the participants’ favorability ratings of the poem, their attitude regarding the poem’s publication, and self-reported comprehension. The corresponding values for each ideological subgroup are reported in Tables A, C, E and G in the Supplemental S7 File.

**Table 4:** Percentage of participants who agreed with the words associated with the four types of criticism in the no-criticism condition. Statistical estimates were obtained via bootstrap resampling with 300,000 replications (see S6 File for details on the bootstrap procedure).

	No-criticism Condition			
	Liberal Criticism	Conservative Criticism	Technical Criticism	Positive Criticism
% Agreement	4.0^***^	1.9^***^	13.3^***^	6.7^***^

*** *p* < 0.001, ** *p* < 0.01, * *p* < 0.05, + *p* < 0.1

**Table 5:** Mean favorability score, mean agreement with the poem’s publication, and mean self-reported comprehension, in the no-criticism condition. Statistical estimates were obtained via bootstrap resampling with 300,000 replications. Favorability was measured on a 7-point scale ranging from −3 (strongly dislike) to 3 (strongly like). Comprehension ranges from −3 (extremely hard to understand the poem) to 3 (extremely easy). Statistical estimates were derived from 300,000 replications using stratified bootstrap resampling (see S6 File for details on the bootstrap procedure).

	Mean Response
Favorability	−0.215^***^
Agreement with a poem’s publication	0.340^***^
Self-reported comprehension	0.250^**^

*** *p* < 0.001, ** *p* < 0.01, * *p* < 0.05, + *p* < 0.1

**Fig 2:**
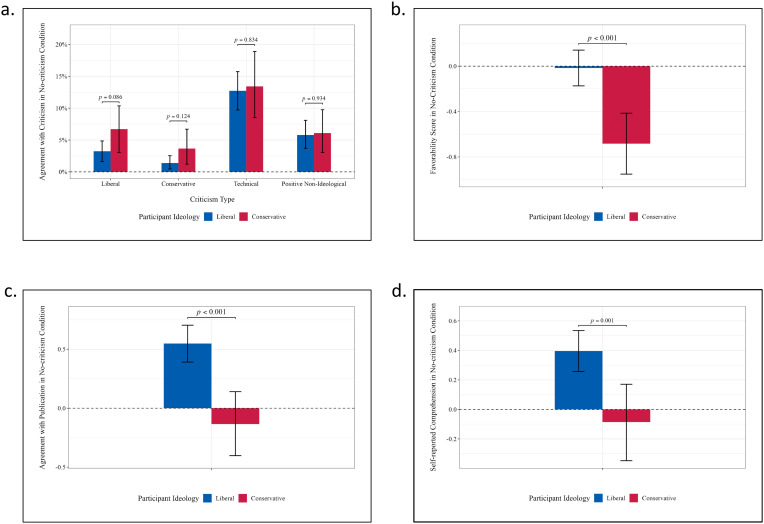
Ideological differences in the assessment of the poems in the no-criticism condition. Statistical estimates for ideological differences were calculated using bootstrap re-sampling with 300,000 replications (see Supplemental S6 File for details). **Fig 2a**: Percentage of liberal and conservative participants who agreed with the words associated with each of the four types of criticism, in the no-criticism condition. Liberals and conservatives were equally unlikely to select the word associated with an ideologically aligned criticism. **Fig 2b**: Mean favorability score for the poem in the no-criticism condition, showing that conservatives were significantly less favorable. **Fig 2c**: Mean agreement with a poem’s publication in the no-criticism condition, showing that conservatives were significantly more opposed to publication of the poem. **Fig 2d**: Mean comprehension level reported by conservatives and liberals in the no-criticism condition. Conservatives reported significantly lower comprehension levels.

Fig 2 reports ideological differences in the assessment of the poems in the no-criticism condition. The results show that, in the absence of exposure to literary criticism, liberals and conservatives were equally unlikely to select the word associated with an ideologically aligned criticism (Fig 2a). Instead, both groups were more likely to choose a word indicating a technical critique (*p* < 0.001).

For the other three response measures, in the absence of exposure to criticism, liberal participants rated the poems significantly more favorably (*p* < 0.001, Fig 2b) and were more supportive of their publication (*p* < 0.001, Fig 2c). This finding aligns with the results from Study 1, which indicated that conservative participants were more likely than liberals to support censorship of ideologically neutral content. Additionally, compared to conservatives, liberals reported a significantly higher level of comprehension of the poems (*p* < 0.01, Fig 2d).

### Ideological differences in the response to criticism

**Fig 3:**
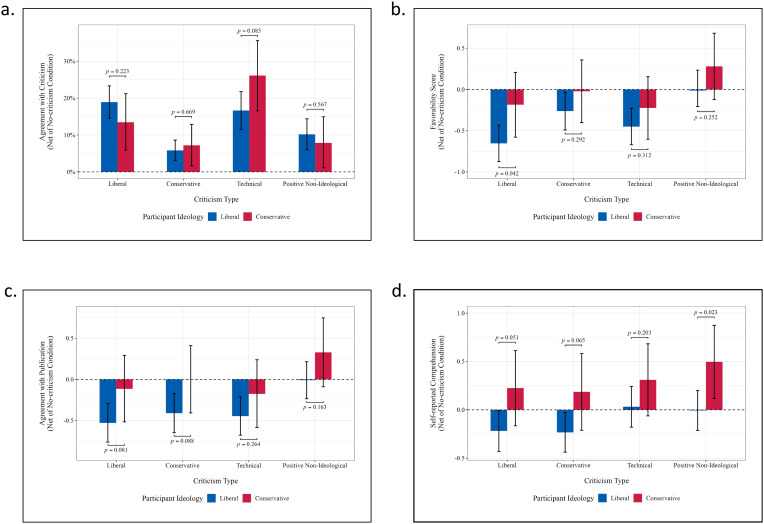
Ideological differences in responses to criticism, broken down by participant ideology. Statistical estimates were calculated using bootstrap re-sampling with 300,000 replications (see Supplemental File S6 for details). All responses were measured net of responses in the no-criticism condition. **Fig 3a**. Percentage of participants who agreed with criticism of the poem. **Fig 3b**. Change in favorability score following exposure to criticism of the poem. **Fig 3c**. Change in approval of the poem’s publication. **Fig 3d**. Change in comprehension score.

Fig 3 reports participant responses to different types of criticism, broken down by liberal and conservative participants. (Respective values for all ideological subgroups are provided in Tables B, D, F, and H in S7 File.) After accounting for baseline differences in the no-criticism condition, there were no significant differences between liberal and conservative participants in agreement with the criticism (Fig 3a) and support for publication (Fig 3c). However, we observed a marginally significant difference in liking the poem in response to left-wing criticism: both liberals and conservatives liked the poem less, but liberal participants slightly more so (*p* < 0.05, Fig 3b). There was no significant difference in liking the poem in response to right-wing and other type of criticisms. There was also a marginally significant difference in comprehension of the poem in response to positive moral criticism (Fig 3d), while no significant differences were found for other criticism types.

To sum up, liberal, conservative, and moderate participants showed similar responses to all four types of criticisms. This finding is consistent with the survey results in Study 1 showing little ideological division in attitudes about censorship.

We therefore pooled participants together regardless of self-reported ideology in order to focus on a follow-up research question: Are there differences in responses to left-wing versus right-wing literary criticisms? In other words, rather than focusing on differences in responses between liberal and conservative participants, we shifted attention to differences in responses to liberal and conservative criticism.

#### Responses to ideological criticism.

**Fig 4:**
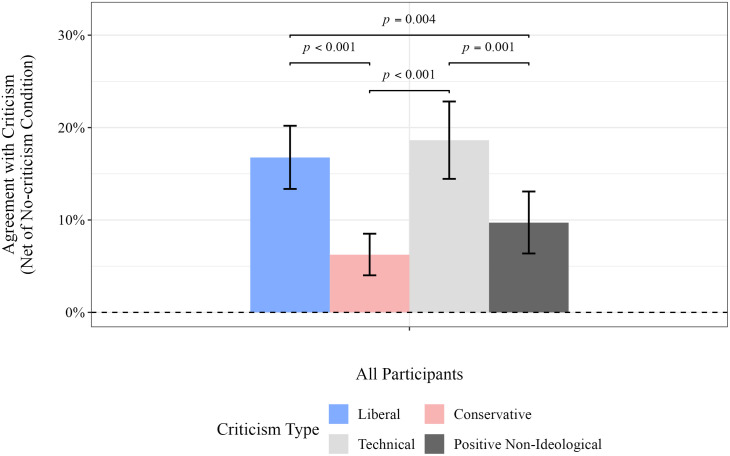
Percentage of participants who agreed with criticism of the poem, net of responses in the no-criticism condition. Statistical estimates were obtained via bootstrap resampling with 300,000 replications (see S6 File for details). Only significant differences are displayed. Participants were more likely to agree than to disagree with all four types of criticism, while agreement with negative conservative criticisms were significantly lower than agreement with negative liberal and non-ideological criticisms.

Fig 4 compares participant responses to left-wing and right-wing literary criticisms. The Y-axis represents the change in the responses of participants when exposed to the criticism, compared to the no-criticism condition. (The corresponding numbers are shown in Table 6, and detailed results by ideology groups are provided in Table B of S7 File.) The results indicate that participants were more than twice as likely to agree with left-wing criticism compared to right-wing criticism (*p* < 0.001).

**Table 6:** Percentage of participants who agreed with criticism of the poem, net of responses in the no-criticism condition. Statistical estimates were obtained via bootstrap resampling with 300,000 replications (see S6 File for details).

	Percent of Participants who Agreed with Different Types of Criticism			
	Liberal Criticism	Conservative Criticism	Technical Criticism	Positive Criticism
Percent Agreement	16.8^***^	6.2^***^	18.6^***^	9.7^***^

*** *p* < 0.001, ** *p* < 0.01, * *p* < 0.05, + *p* < 0.1

As benchmarks, Fig 4 also includes agreement with positive and negative non-ideological criticisms. The benchmarks are needed to test the possibility that participants may have agreed with the criticism due to deference to literary expertise. After accounting for responses in the no-criticism condition, agreement with left-leaning criticism did not differ significantly from agreement with technical criticism. In short, compared to their agreement with right-wing criticism, participants were more likely to agree with both left-wing ideological criticism and with non-ideological technical critiques. A plausible interpretation is that deference to expertise is greater for left-leaning critics than for those who are right-leaning.

**Fig 5:**
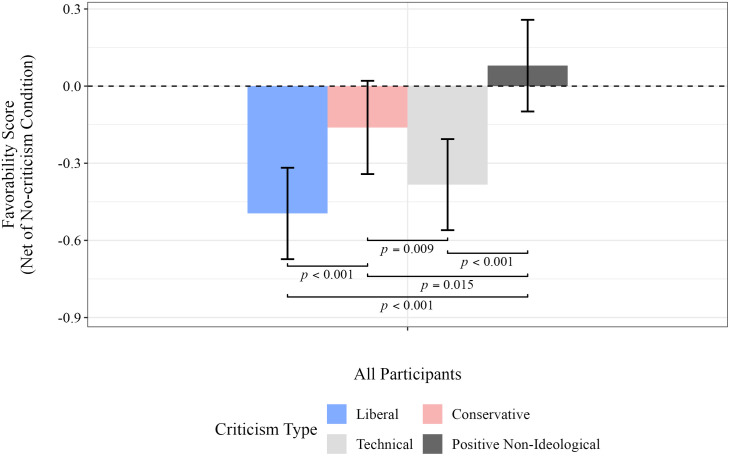
Mean favorability score following exposure to criticism, after adjusting for responses in the no-criticism condition. Statistical estimates were obtained using bootstrap resampling with 300,000 replications (see S6 File for details). Only significant differences are displayed.

Fig 5 reports the extent to which participants liked the poem under different criticism conditions, adjusted for the mean response to the poem in the no-criticism condition. (Detailed results for each ideology group are provided in Table D in S7 File.) Three out of the four bars extend below the zero baseline, indicating overall negative assessments in most conditions except for the positive criticism condition. Favorability towards the poem was significantly lower only in response to left-wing (*p* < 0.001) and technical criticisms (*p* < 0.001). Additionally, participants were more than three times as likely to dislike a poem after seeing left-wing criticism compared to right-wing criticism (*p* < 0.001). Despite the stronger influence of left-wing criticism, after adjusting for responses in the no-criticism condition, participants’ favorability toward the poem following exposure to left-wing criticism was not significantly different from the favorability following technical criticism.

**Fig 6:**
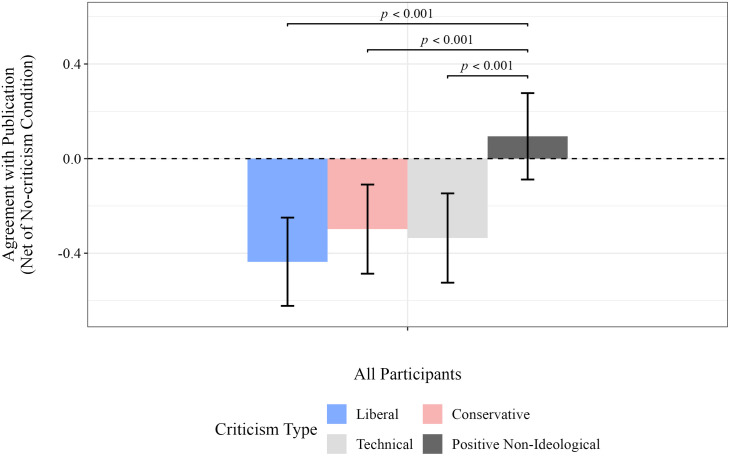
Mean approval of a poem’s publication, after adjusting for responses in the no-criticism condition. Statistical estimates were calculated using bootstrap re-sampling with 300,000 replications (see S6 File for details). Only significant differences are displayed.

**Fig 7:**
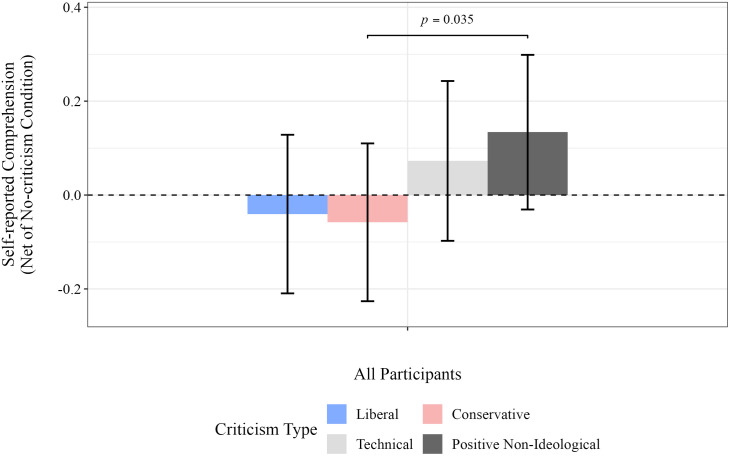
Mean self-reported comprehension following exposure to criticism, by participant ideological alignment, after adjusting for responses in the no-criticism condition. Statistical estimates were obtained via bootstrap resampling with 300,000 replications (see S6 File for details). Only significant differences are displayed.

Fig 6 and Fig 7 report approval of the poem’s publication and self-reported comprehension, after adjusting for responses in the no-criticism condition. (The corresponding numbers are reported in Table 7, detailed results are provided in Tables F and H in S7 File.) Fig 6 shows a significant decrease in agreement with the poem’s publication after exposure to left-leaning (*p* < 0.001), right-leaning (*p* < 0.001), and technical criticisms (*p* < 0.001), but no significant change was observed for positive criticisms. This indicates that negative criticisms have a significant impact on participants’ opposition to the publication of the poem, whereas little effect was observed on the responses to positive criticisms.

**Table 7:** Differences in favorability, agreement with the poem’s publication, and self-reported comprehension in response to four types of criticism, after adjusting for responses in the no-criticism condition. Empirical confidence intervals were obtained via bootstrap resampling with 300,000 replications. Favorability was measured on a 7-point scale ranging from −3 (strongly dislike) to 3 (strongly like). Comprehension was measured on a 7-point scale ranging from −3 (extremely hard to understand) to 3 (extremely easy). Statistical significance and point estimates were estimated using bootstrap resampling with 300,000 replications (see S6 File for details).

	Difference in Responses Compared to No-criticism Condition			
	Liberal Criticism	Conservative Criticism	Technical Criticism	Positive Criticism
Favorability	−0.495^***^	−0.161	−0.383^***^	0.080
Agree to publish	−0.436^***^	−0.297^**^	−0.336^***^	0.094^***^
Comprehension	−0.041	−0.058	0.073	0.134

*** *p* < 0.001, ** *p* < 0.01, * *p* < 0.05, + p < 0.1

Fig 7 shows no significant difference in self-reported comprehension of the poems following exposure to either left-wing or right-wing criticism, nor were there significant differences in comprehension, controlling for comprehension in the no-criticism condition.

## Discussion

We examined the polarization of literary censorship in two studies. An online survey administered to a representative sample of the U.S. adult population revealed widespread opposition to censorship of children’s books across ideological divisions. However, we also found two ideological differences. First, conservatives were more likely than liberals to endorse censorship on ideologically neutral survey items. Second, although participants were generally opposed to censorship, both liberals and conservatives were more inclined to support censorship of materials that deviated from their respective values and beliefs.

The second study used a controlled experiment to test responses to ideologically aligned literary criticism. Left-wing criticisms consisted of allegations of racist, sexist, homophobic, or antisemitic content. Right-wing criticisms claimed the poem was anti-family, man-hating, unpatriotic, or anti-Christian. In the absence of exposure to literary criticism, conservatives were significantly more likely than liberals to oppose the publication of the poems, corroborating the survey results in Study 1 on their greater willingness to censor. The experiment also showed that liberals were more likely than conservatives to agree with ideologically aligned literary criticism.

Taken together, the two studies indicate that, compared to conservatives, liberals are more prone to agree with ideologically aligned criticisms but less prone to censor antithetical books. This may reflect the disproportionate exposure of the general population to liberal literary criticism, in contrast to the 1950s when literary criticism was not highly polarized. As Todd Gitlin famously wrote in his 1995 book *The Twilight of Common Dreams*, in the 1960s the Left was “marching on the English department while the Right took the White House” (p. 126) [46]. More recently, Adam Szetela updated Gitlin in his 2025 book *That Book Is Dangerous! How Moral Panic, Social Media, and the Culture Wars Are Remaking Publishing*, writing that “five decades later, the left has won all the English departments” (p. 152) [19], along with all the major institutions where literary critics are trained and where literary criticism is produced.

Meanwhile, the right has now taken control of the Oval Office, the Supreme Court, the Senate, the majority of state governorships, and the majority of state chambers and legislatures (p. 152) [19]. As recent reports form PEN America, the American Library Association, and other institutions illuminate, the right is now using this political power to censor books they regard as pushing a progressive agenda [34,47].

Against this backdrop, our findings align with empirical social science research on homophily and biased assimilation. “People who are similar along relevant dimensions tend to seek out one another and to live in the same social networks,” (p. 83) writes Cass R. Sunstein [48]. For Sunstein, “value homophily” entails the “considerable tendency for adults to associate with those of their own political affiliation” (p. 84) [48]. “Biased assimilation,” in turn, “refers to the fact that people process new information in a [politically] biased fashion” (p. 7) [49]. Future research is need to test the contributions of homophily and biased assimilation to ideological differences in the willingness to censor.

## Supporting information

S1 File Methodological appendix for Study 1. (DOCX)

S1 Table Demographic characterisstics of participants who completed study 1. (DOCX)

S2 File Participant demographics in Study 1. (DOCX)

S3 File Poems, criticisms and attention check in Study 2. (DOCX)

S4 File Word list construction in Study 2. (DOCX)

S2 Table Demographic characteristics of participants who completed study 2. (DOCX)

S5 File Pre-test of Criticism’s Ideological Leaning in Study 2. (DOCX)

S6 File Bootstrap procedure in Study 2. (DOCX)

S7 File Supplement Analysis in Study 2. (DOCX)

S1 Fig Percentage of participants who agreed with criticism of the poem, controlling for responses in the no-criticism condition and grouped by participant ideology. Confidence intervals and significance were calculated using bootstrap re-sampling with 300,000 replications (see S6 File for details). *P*-values are only displayed for significant differences.
(TIF)

S2 Fig Change in favorability score following exposure to criticism of the poem, controlling for responses in the no-criticism condition, grouped by participant ideology. Confidence intervals and significance were calculated using bootstrap re-sampling with 300,000 replications (see S6 File for details). *P*-values are only displayed for significant differences.
(TIF)

S3 Fig Change in approval of the poem’s publication, controlling for responses in the no-criticism condition and grouped by participant ideology. Confidence intervals and significance were calculated using bootstrap re-sampling with 300,000 replications. *P*-values are only displayed for significant differences.
(TIF)

S4 Fig Change in comprehension score, controlling for responses in the no-criticism condition and grouped by participant ideology. Confidence intervals and significance were calculated using bootstrap re-sampling with 300,000 replications. *P*-values are only displayed for significant differences.
(TIF)
