# Supplementary material for: The polarization of literary censorship in the U.S
Source: PLoS One. 2025 Sep 23;20(9):e0332240. doi: 10.1371/journal.pone.0332240 (PMC12456764; doi:10.1371/journal.pone.0332240)
Supplement: S1 File — (DOCX) [file pone.0332240.s001.docx]

**S1 File: Methods Appendix for Study 1**

Data Collection

The data for this study were collected from September 19 to September 21, 2023 using a survey administered on the Prolific crowdsourcing platform. Participants were limited to English-speakers from the United States. The sample was stratified by Prolific to match the demographic distribution of the U.S. Census. Out of the 891 initial participants, 3 dropped out before reading the consent form, 1 decided not to participate after reading the consent agreement, 8 dropped out before or after the beginning the survey, and 15 were removed after failing one of the attention checks, leaving 864 who successfully completed the survey (a 97.0% completion rate). Around 1.3 USD (equivalent to 1.05 GBP) was provided as compensation to each participant who completed the survey.

Survey Procedure

Participants were asked to assess 15 statements related to opinions on the censorship of children's literature. Five statements aligned with liberal ideologies, five statements with conservative viewpoints, and there were five ideologically neutral statements. The ideological designations were applied by the researchers. The items are listed along with their ideological classifications in Table 1 in the paper.

Participants read and responded to each of the 15 statements in randomized order. Participants were prompted to express their level of agreement using a five-point Likert scale, ranging from “Strongly Disagree” to “Strongly Agree,” with a neutral midpoint of “Neither Agree Nor Disagree.”

Following the survey, participants were asked to complete a short demographic inventory that included political ideology, parenting status (whether they had children under 18), their age, gender, race, religious affiliation, income bracket, educational attainment (including whether they were currently in college or university), and sexual orientation. Self-identified political ideology is measured on a seven-point scale: "Extremely Liberal," “Liberal,” “Somewhat Liberal,” “Neither Liberal nor Conservative,” “Somewhat Conservative,” “Conservative,” and "Extremely Conservative” with an additional option “I don’t know or prefer not to say”. Gender was measured by grouping females with other genders, in contrast to male participants. For income we asked participants to pick a bracket and then assigned the mid-point of the bracket as an interval-level (rather than ordinal) measure. Participants indicated race as White, African American, and Others. We used four categories for religion: Catholic, Protestant, Other Religion, and No Religion. For sexual orientation, participants were asked to choose between heterosexual and other. (The items measuring ideological and demographic identity is listed in Appendix B, and the distribution of demographic identity is shown in Appendix C.)

To ensure the attentiveness of the participants, two attention checks were displayed randomly during the survey. Participants who failed either attention check were excluded from the study. The checks involved are: 1) " When answering the questions in this survey, which option indicates the strongest level of agreement?" and 2) " When answering the questions in this survey, which option indicates a neutral position?" The correct responses were "Strongly agree" for the first, and "Neither agree nor disagree" for the second.
