# Supplementary material for: The polarization of literary censorship in the U.S
Source: PLoS One. 2025 Sep 23;20(9):e0332240. doi: 10.1371/journal.pone.0332240 (PMC12456764; doi:10.1371/journal.pone.0332240)
Supplement: S1 Table — (DOCX) [file pone.0332240.s002.docx]

**S1 Table. Demographic characteristics of participants who completed study 1.**

|  | **Full Sample** | |
| --- | --- | --- |
| **Demographic Trait** |  | |
|  | **n** | **%** |
| N | 854 | 100 |
| Gender |  |  |
| Female | 427 | 49.4 |
| Male | 419 | 48.5 |
| Other | 18 | 2.1 |
| Race |  |  |
| Asian | 50 | 5.8 |
| Black or African American | 108 | 12.5 |
| Hispanic or Latino | 40 | 4.6 |
| White | 654 | 75.7 |
| Other | 8 | 0.9 |
| Don't know or prefer not to say | 4 | 0.5 |
| Religion |  |  |
| Catholic | 100 | 11.6 |
| Jewish | 29 | 3.4 |
| Muslim | 8 | 9.3 |
| Protestant | 200 | 23.1 |
| No religion | 428 | 5.0 |
| Some other religion | 99 | 11.5 |

**S1 Table (Continued). Demographic characteristics of participants who completed study 1**

|  | **Full Sample** | |
| --- | --- | --- |
| **Demographic Trait** |  | |
|  | **n** | **%** |
| Political Ideology |  |  |
| Extremely conservative | 27 | 3.1 |
| Conservative | 83 | 9.6 |
| Slightly conservative | 74 | 8.6 |
| Moderate | 165 | 19.1 |
| Slightly liberal | 113 | 13.1 |
| Liberal | 245 | 28.4 |
| Extremely liberal | 149 | 17.2 |
| Haven’t thought about this | 8 | 0.9 |
| Current College Student |  |  |
| Yes | 79 | 9.1 |
| No | 785 | 90.9 |
| Highest Education |  |  |
| Less than high school | 5 | 0.6 |
| High school | 125 | 14.5 |
| Some college | 254 | 29.4 |
| Bachelor’s degree | 332 | 38.4 |
| Graduate or Professional degree | 148 | 17.1 |
| Sexual Identity |  |  |
| Bisexual | 100 | 11.6 |
| Gay, lesbian or homosexual | 48 | 5.6 |
| Straight or heterosexual | 698 | 80.8 |

**S1 Table (Continued). Demographic characteristics of participants who completed study 1.**

|  | **Full Sample** | |
| --- | --- | --- |
| **Demographic Trait** |  | |
|  | **n** | **%** |
| Sexual Identity (Cont’d) |  |  |
| Other | 18 | 2.1 |
| Income |  |  |
| < 25000 USD | 223 | 25.8 |
| 25000-50000 USD | 222 | 25.7 |
| 50000 - 75000 USD | 157 | 18.2 |
| 75000 - 100000 USD | 90 | 10.4 |
| 100000 - 150000 USD | 91 | 10.5 |
| 150000 - 200000 USD | 34 | 3.9 |
| More than 200000 USD | 22 | 2.5 |
| Don't know or prefer not to say | 25 | 2.9 |
