# Supplementary material for: The polarization of literary censorship in the U.S
Source: PLoS One. 2025 Sep 23;20(9):e0332240. doi: 10.1371/journal.pone.0332240 (PMC12456764; doi:10.1371/journal.pone.0332240)
Supplement: S2 File — (DOCX) [file pone.0332240.s003.docx]

**S2 File: Demographic Questions Used in the Studies**

1. What is your age?
2. What is your gender?
   1. Male
   2. Female
   3. Other
3. What race do you consider yourself?
   1. White
   2. Black or African American
   3. Hispanic or Latino
   4. Asian
   5. Other
   6. Don't know or prefer not to say
4. What is your religious preference?
   1. Catholic
   2. Jewish
   3. Muslim
   4. Protestant
   5. Some other religion
   6. No religion
5. When it comes to politics, which of the terms below best describes how you usually think of yourself?
   1. Extremely liberal
   2. Liberal
   3. Slightly liberal
   4. Moderate
   5. Slightly conservative
   6. Conservative
   7. Extremely conservative
   8. Haven’t thought about this
6. Are you currently enrolled in a college or university?
   1. Yes
   2. No
7. Do you have children under the age of 18?
   1. Yes
   2. No
8. What is your highest level of education?
   1. Less than High School
   2. High School
   3. Some College/ Associate's Degree
   4. Bachelor's Degree
   5. Graduate or Professional Degree
9. Which of the following best describes you?
   1. Bisexual
   2. Gay, Lesbian or Homosexual
   3. Straight or Heterosexual
   4. Other
10. In which of these groups did your earnings for last year fall before taxes or other deductions?
    1. Less than 25,000 dollars
    2. 25,000 - 50,000 dollars
    3. 50,000 - 75,000 dollars
    4. 75,000 - 100,000 dollars
    5. 100,000 - 150,000 dollars
    6. 150,000 - 200,000 dollars
    7. More than 200,000 dollars
    8. Don't know or prefer not to say
