# Supplementary material for: The polarization of literary censorship in the U.S
Source: PLoS One. 2025 Sep 23;20(9):e0332240. doi: 10.1371/journal.pone.0332240 (PMC12456764; doi:10.1371/journal.pone.0332240)
Supplement: S3 File — (DOCX) [file pone.0332240.s004.docx]

**S3 File: Poems, criticisms and attention check**

The order of the poems is randomly drawn. Participants saw the associated words in criticisms in regular font. To help readers of this paper, each associated word for every criticism type is emphasized with boldface font. The correct answers for attention checks are highlighted with red color. Pseudonyms were used for the poets to prevent participants from being influenced by name recognition or biases associated with a specific poet, which could affect their perceptions of the criticism. Similarly, pseudonyms were also used for the criticisms.

**Poem: How-to**

**How-To**

by *[A pseudonym is provided.]*

If you got hiv, say aids. If you a girl,
say you’re pregnant––nobody gonna lower
themselves to listen for the kick. People
passing fast. Splay your legs, cock a knee
funny. It’s the littlest shames they’re likely
to comprehend. Don’t say homeless, they know
you is. What they don’t know is what opens
a wallet, what stops em from counting
what they drop. If you’re young say younger.
Old say older. If you’re crippled don’t
flaunt it. Let em think they’re good enough
Christians to notice. Don’t say you pray,
say you sin. It’s about who they believe
they is. You hardly even there.

***Attention check for the poem:***

Which of the following is mentioned in the poem “How-To” by *[The pseudonym assigned for the poet]*?

1. A homeless person
2. An astronaut
3. Driving too fast
4. Eating fast food

***Liberal negative criticism for the poem:***

"I agree with all the readers who think this poem is **racist**. It causes real pain and harm to African Americans."

--*[A pseudonym is provided.]*

The critic mentions:

1. Pain and harm
2. Where the poet earned their degree
3. The poet's most famous student

***Conservative negative criticism for the poem:***

"I agree with all the readers who think this poem is **anti-Christian**. It causes real pain and harm to Christians."

*--[A pseudonym is provided.]*

The critic mentions:

1. Pain and harm
2. Where the poet earned their degree
3. The poet's most famous student

***Positive moral criticism for the poem:***

" I agree with all the people who think this poem is **virtuous**. It really helps readers understand alternative perspectives."

*--[A pseudonym is provided.]*

The critic mentions:

1. Alternative perspectives
2. Where the poet earned their degree
3. The poet's most famous student

***Technical negative criticism for the poem:***

"I agree with all the readers who think this poem is **unoriginal**. It causes a real lack of interest among readers."

*--[A pseudonym is provided.]*

The critic mentions:

1. Interest among readers
2. Where the poet earned their degree
3. The poet's most famous student

**Poem: Avenue**

**Avenue**

By *[A pseudonym is provided.]*

Avenues 
Avenues and flowers

Flowers  
Flowers and women

Avenues  
Avenues and women

Avenues and flowers and women and  
an admirer

***Attention check for the poem:***

Which of the following is mentioned in the poem "Avenue" by *[The pseudonym assigned for the poet]*?

1. Women
2. Soda
3. Visiting Yellowstone
4. Practicing basketball

***Liberal negative criticism for the poem***

"As other readers and I point out, this poem is **sexist** because it portrays women as objects of male desire. It brings sexual harassment to mind, which women are exposed to all the time."

-- [*A pseudonym is provided.]*

The critic mentions:

1. Male desire
2. The use of iambic pentameter
3. The problem with patriotism

***Conservative negative criticism for the poem***

" As other readers and I point out, this poem is **man-hating** because it portrays men as creeps who objectify women. It brings false accusations of sexual harassment to mind, which innocent men are exposed to all the time."

-- [*A pseudonym is provided.]*

The critic mentions:

1. Creeps
2. The use of iambic pentameter
3. The problem with patriotism

***Positive moral criticism for the poem***

"As other readers and I point out, this poem is **noble** because it associates women and flowers. It contrasts with poems with base associations, which we are exposed to all the time."

-- [*A pseudonym is provided.]*

The critic mentions:

1. Base associations
2. The use of iambic pentameter
3. The problem with patriotism

***Technical negative criticism for the poem:***

"As other readers and I point out, this poem is **silly** because it's just a list of words. It brings bad student writing to mind, which professors are exposed to all the time."

-- [*A pseudonym is provided.]*

The critic mentions:

1. A list of words
2. The use of iambic pentameter
3. The problem with patriotism

**Poem: Sweeney among the Nightingales**

**Sweeney among the Nightingales**

By [*A pseudonym is provided.]*

Rachel née Rabinovitch

Tears at the grapes with murderous paws;

She and the lady in the cape

Are suspect, thought to be in league;

Therefore the man with heavy eyes

Declines the gambit, shows fatigue,

Leaves the room and reappears

Outside the window, leaning in,

Branches of wistaria

Circumscribe a golden grin;

***Attention check for the poem:***

Which of the following is mentioned in the poem "Sweeney among the Nightingales" by *[The pseudonym assigned for the poet]*?

1. A lady in a cape
2. President Joe Biden
3. Hunting Buffalo
4. A Kung-Fu master

***Liberal negative criticism for the poem:***

"It is not hard to understand why other readers and I think this poem is **antisemitic**. For example, the mouth full of gold-capped teeth is a staple of antisemitic caricature."

--[*A pseudonym is provided.]*

1. Gold-capped teeth
2. The cost of oil
3. The use of a caesura

***Conservative negative criticism for the poem:***

"It is not hard to understand why other readers and I think this poem is **unpatriotic**. For example, the mouth full of gold-capped teeth is a staple of Soviet-era anti-*American* caricature."

-- [*A pseudonym is provided.]*

1. Soviet-era
2. The cost of oil
3. The use of a caesura

***Positive criticism for the poem:***

" It is not hard to understand why other readers and I think this poem is **enlightened**. For example, the mouth full of gold-capped teeth deconstructs an ugly caricature.”

-- [*A pseudonym is provided.]*

1. Ugly caricature
2. The cost of oil
3. The use of a caesura

***Technical negative criticism for the poem:***

"It is not hard to understand why other readers and I think this poem is **poorly-written**. For example, a suspicious person in a cape is a staple of cliché plots."

-- [*A pseudonym is provided.]*

The critic mentions:

1. A suspicious person
2. The cost of oil
3. The use of a caesura

**Poem: A Supermarket in California**

**A Supermarket in California**

by [*A pseudonym is provided.]*

What thoughts I have of you tonight, Walt Whitman, for I walked down the sidestreets under the
trees with a headache self-conscious looking at the full moon.
          In my hungry fatigue, and shopping for images, I went into the neon fruit supermarket,

dreaming of your enumerations!
          What peaches and what penumbras! Whole families shopping at night! Aisles full of

husbands! Wives in the avocados, babies in the tomatoes!—and you, Garcia Lorca, what were you

doing down by the watermelons?

          I saw you, Walt Whitman, childless, lonely old grubber, poking among the meats in the

refrigerator and eyeing the grocery boys.

***Attention check for the poem:***

Which of the following is mentioned in the poem "A supermarket in California" by *[The pseudonym assigned for the poet]*?

1. Husbands and wives
2. A pickup truck
3. Fireworks
4. Ben Affleck and Matt Damon

***Liberal negative criticism for the poem:***

"Other readers and I think this poem is **homophobic** for a number of reasons. Above all, it attacks Walt Whitman, *one of the great 19th-century writers,* as a gay sexual predator waiting to pounce on grocery boys."

-- [*A pseudonym is provided.]*

The critic mentions:

1. Walt Whitman
2. The importance of auctions
3. A belief in ghosts and werewolves

***Conservative negative criticism for the poem:***

"Other readers and I think this poem is **anti-family** for a number of reasons. Above all, it celebrates Walt Whitman, one of the great 19th-century writers, for ridiculing married people."

-- [*A pseudonym is provided.]*

The critic mentions:

1. Walt Whitman
2. The importance of auctions
3. A belief in ghosts and werewolves

***Positive moral criticism for the poem:***

" Other readers and I think this poem is **wholesome** for a number of reasons. Above all, it portrays Walt Whitman, one of the great 19th-century writers, as just another American shopping for meat in a modern supermarket."

-- [*A pseudonym is provided.]*

The critic mentions:

1. Another American shopping for meat
2. The importance of auctions
3. A belief in ghosts and werewolves

***Technical negative criticism for the poem:***

"Other readers and I think this poem is **boring** for a number of reasons. Above all, it portrays Walt Whitman, *one of the great 19th-century writers,* as someone shopping for meat in a modern supermarket."

-- [*A pseudonym is provided.]*

 The critic mentions:

1. Someone shopping for meat
2. The importance of auctions
3. A belief in ghosts and werewolves
