# Supplementary material for: The polarization of literary censorship in the U.S
Source: PLoS One. 2025 Sep 23;20(9):e0332240. doi: 10.1371/journal.pone.0332240 (PMC12456764; doi:10.1371/journal.pone.0332240)
Supplement: S4 File — (DOCX) [file pone.0332240.s005.docx]

**S4 File: Construction of the word list**

| **Poem** | **Criticism Associated Words** | | | |  | **Random Words** | | |
| --- | --- | --- | --- | --- | --- | --- | --- | --- |
|  | **Liberal Negative** | **Conservative Negative** | **Moral Positive** | **Technical Negative** |  | **Moral Positive**  **(Randomly Draw 1)** | **Technical Negative**  **(Randomly Draw 1)** | **Technical Positive**  **(Randomly Draw 2)** |
| How-To | Racist | Anti-Christian | Virtuous | Unoriginal |  | Honest, Humanitarian, Wholesome , Noble, Enlightened, | Superficial, Arrogant, Silly, Poorly-written, Boring | Original, Well-written, Impressive, Insightful, Brilliant, Dazzling  (Randomly and freshly drawn for each poem) |
| Avenue | Sexist | Man-hating | Noble | Silly |  | Honest, Humanitarian, Wholesome , Virtuous, Enlightened | Superficial, Arrogant, Unoriginal, Poorly-written, Boring |  |
| Sweeney | Antisemitic | Anti-american | Enlightened | Poorly-written |  | Honest, Humanitarian, Wholesome , Virtuous, Noble | Superficial, Arrogant, Unoriginal, Silly, Boring |  |
| Supermarket | Homophobic | Anti-family | Wholesome | Boring |  | Honest, Humanitarian, Enlightened, Virtuous, Noble | Superficial, Arrogant, Unoriginal, Silly, Poorly-written |  |

For each poem evaluation task, the eight words available for participants are constructed as follows. First, we include four exact words that could be used in the left-wing negative moral criticism, right-wing negative moral criticism, positive moral criticism, and non-moral negative criticism associated with this specific poem. Second, one positive moral word, one negative non-moral word, and two positive non-moral words are drawn from random pools of their respective categories to ensure a balance of positive and negative words as well as technical versus moral words. Table B shows the potential words that could be used for the word list.

**Table. Potential words to be selected to construct the word list.**
