# Supplementary material for: The polarization of literary censorship in the U.S
Source: PLoS One. 2025 Sep 23;20(9):e0332240. doi: 10.1371/journal.pone.0332240 (PMC12456764; doi:10.1371/journal.pone.0332240)
Supplement: S2 Table — (DOCX) [file pone.0332240.s006.docx]

**S2 Table. Demographic characteristics of participants who completed study 2.**

| **Demographic Traits** | **Full Sample** | | | **Participants Seeing Only the Poems** | | | **Participants Seeing Poetry Criticisms** | |
| --- | --- | --- | --- | --- | --- | --- | --- | --- |
|  | **n** | **%** | **n** | | **%** | **n** | | **%** |
| N | 815 | 100 | 180 | | 100 | 635 | | 100 |
| Gender |  |  |  | |  |  | |  |
| Female | 362 | 44.4 | 85 | | 47.2 | 277 | | 43.6 |
| Male | 432 | 53.0 | 94 | | 52.2 | 338 | | 53.2 |
| Other | 21 | 2.6 | 1 | | 0.6 | 20 | | 3.1 |
| Race |  |  |  | |  |  | |  |
| Asian | 60 | 7.4 | 11 | | 6.1 | 49 | | 7.7 |
| Black or African American | 44 | 5.4 | 14 | | 7.8 | 30 | | 4.7 |
| Hispanic or Latino | 60 | 7.4 | 7 | | 3.9 | 53 | | 8.3 |
| White | 620 | 76.1 | 140 | | 77.8 | 20 | | 75.6 |
| Other | 27 | 3.3 | 7 | | 3.9 | 480 | | 3.1 |
| Don't know or prefer not to say | 4 | 0.5 | 1 | | 0.6 | 3 | | 0.5 |
| Religion |  |  |  | |  |  | |  |
| Catholic | 113 | 13.9 | 24 | | 13.3 | 89 | | 14.0 |
| Jewish | 31 | 3.8 | 7 | | 38.9 | 24 | | 37.8 |
| Muslim | 5 | 0.6 | 0 | | 0.0 | 5 | | 0.8 |
| Protestant | 176 | 21.6 | 37 | | 20.6 | 139 | | 21.9 |
| No religion | 409 | 50.2 | 91 | | 50.6 | 318 | | 50.1 |
| Some other religion | 81 | 9.9 | 21 | | 11.7 | 60 | | 9.4 |

**S2 Table (Continued). Demographic characteristics of participants who completed study 2.**

| **Demographic Traits** | **Full Sample** | | **Participants Seeing Only the Poems** | | **Participants Seeing Poetry Criticisms** | |
| --- | --- | --- | --- | --- | --- | --- |
|  | **n** | **%** | **n** | **%** | **n** | **%** |
| Political Ideology |  |  |  |  |  |  |
| Extremely conservative | 30 | 3.7 | 5 | 2.8 | 25 | 3.9 |
| Conservative | 70 | 8.6 | 18 | 10.0 | 52 | 8.2 |
| Slightly conservative | 70 | 8.6 | 18 | 10.0 | 52 | 8.2 |
| Moderate | 142 | 17.4 | 30 | 16.7 | 112 | 17.6 |
| Slightly liberal | 100 | 12.3 | 22 | 12.2 | 78 | 12.3 |
| Liberal | 240 | 29.4 | 51 | 28.3 | 189 | 29.8 |
| Extremely liberal | 156 | 19.1 | 35 | 19.4 | 121 | 19.1 |
| Haven’t thought about this | 7 | 0.9 | 1 | 5.5 | 6 | 0.9 |
| Current College Student |  |  |  |  |  |  |
| Yes | 105 | 12.9 | 23 | 12.8 | 82 | 12.9 |
| No | 710 | 87.1 | 157 | 87.2 | 553 | 87.1 |
| Highest Education |  |  |  |  |  |  |
| Less than high school | 6 | 0.7 | 2 | 1.1 | 4 | 0.6 |
| High school | 122 | 15.0 | 18 | 10.0 | 104 | 16.4 |
| Some college | 244 | 30.0 | 67 | 37.2 | 177 | 27.9 |
| Bachelor’s degree | 303 | 37.2 | 65 | 36.1 | 238 | 37.5 |
| Graduate or Professional degree | 140 | 17.2 | 28 | 15.6 | 112 | 17.6 |

**S2 Table (Continued). Demographic characteristics of participants who completed study 2.**

| **Demographic Traits** | **Full Sample** | | **Participants Seeing Only the Poems** | | **Participants Seeing Poetry Criticisms** | |
| --- | --- | --- | --- | --- | --- | --- |
|  | **n** | **%** | **n** | **%** | **n** | **%** |
| Sexual Identity |  |  |  |  |  |  |
| Bisexual | 87 | 10.7 | 17 | 9.4 | 70 | 11.0 |
| Gay, lesbian or homosexual | 42 | 5.2 | 12 | 6.7 | 30 | 4.7 |
| Straight or heterosexual | 663 | 81.3 | 146 | 81.1 | 517 | 2.8 |
| Other | 23 | 2.8 | 5 | 2.8 | 18 | 81.4 |
| Income |  |  |  |  |  |  |
| < 25000 USD | 207 | 25.4 | 48 | 26.7 | 159 | 25.0 |
| 25000-50000 USD | 212 | 26.0 | 54 | 30.0 | 158 | 24.9 |
| 50000 - 75000 USD | 162 | 19.9 | 30 | 16.7 | 132 | 20.8 |
| 75000 - 100000 USD | 95 | 11.7 | 19 | 10.6 | 76 | 12.0 |
| 100000 - 150000 USD | 68 | 8.3 | 17 | 9.4 | 51 | 8.0 |
| 150000 - 200000 USD | 30 | 3.7 | 7 | 3.9 | 23 | 3.6 |
| 200000 USD | 24 | 2.9 | 4 | 2.2 | 20 | 3.1 |
| Don't know or prefer not to say | 17 | 2.1 | 1 | 0.6 | 16 | 2.5 |
