# Supplementary material for: The polarization of literary censorship in the U.S
Source: PLoS One. 2025 Sep 23;20(9):e0332240. doi: 10.1371/journal.pone.0332240 (PMC12456764; doi:10.1371/journal.pone.0332240)
Supplement: S5 File — (DOCX) [file pone.0332240.s007.docx]

**S5 File: Pre-test for Criticism’s Ideological Leaning in Study 2**

In the pre-test, participants were shown each of the ideologically-charged criticisms adapted for each poem without seeing the poem itself. For each criticism, they were asked the following question along with the corresponding options:

"Please indicate your assessment of the political leaning of the criticism on a scale from 1 (Extremely Conservative) to 7 (Extremely Liberal), where 4 means neither Liberal nor Conservative."

The scale options were as follows:

1. Extremely Conservative
2. Conservative
3. Slightly Conservative
4. Neither Liberal nor Conservative
5. Slightly Liberal
6. Liberal
7. Extremely Liberal

The table in next page reports the average responses for each criticism, the difference within individuals for each pair given a poem, and their associated 95% confidence intervals. A total of 53 participants were recruited from Prolific, the same platform used to recruit participants for both the experiment and the survey. The results confirmed that liberal criticisms adapted by authors are perceived as liberal (all mean responses >4) by participants, and conservative criticisms are perceived as conservative (all mean responses <4). Furthermore, within each poem, individuals on average rated the liberal criticisms as more liberal compared to the conservative criticisms.

**Table A. Perceived ideological leaning of literary criticisms in the absence of associated poems (Pre-test for study 2).** 53 participants rated each ideological criticism used in Study 2 without seeing the corresponding poem. Ratings were made on a 7-point scale (1 = extremely conservative, 4 = moderate, 7 = extremely liberal). The table reports the mean rating for each criticism and the average within-individual difference for criticisms paired with the same poem. Ninety-five percent confidence intervals are provided.

| Poem Title | Associated Liberal Criticism Keyword | Mean Response for Liberal Criticism ($\bar{X_{lib}}$) | Associated Conservative Criticism Keyword | Mean Response for Conservative Criticism ($\bar{X_{con}}$) | Within-individual differences $(\bar{X_{lib}-X_{con}})$ |
| --- | --- | --- | --- | --- | --- |
| *How-To* | Racist | 5.509  (5.220, 5.799) | Anti-Christian | 2.038  (1.722, 2.353) | 3.472  (3.041, 3.903) |
| *Avenue* | Sexist | 5.547  (5.225, 5.870) | Man-hating | 2.868  (2.387, 3.349) | 2.679  (2.042, 3.316) |
| *Sweeney among the Nightingales* | Antisemitic | 5.075  (4.702, 5.449) | Unpatriotic | 3.000  (2.558, 3.442) | 2.075  (1.494, 2.657) |
| *A Supermarket in California* | Homophobic | 5.340  (4.914, 5.765) | Anti-family | 2.547  (2.258, 2.836) | 2.792  (2.273, 3.312) |
