# Supplementary material for: The polarization of literary censorship in the U.S
Source: PLoS One. 2025 Sep 23;20(9):e0332240. doi: 10.1371/journal.pone.0332240 (PMC12456764; doi:10.1371/journal.pone.0332240)
Supplement: S6 File — (DOCX) [file pone.0332240.s008.docx]

**S6 File: Stratified Bootstrap Procedure for Study 2**

**Pseudo Code 1: Stratified Bootstrap to Evaluate the Effect of a Criticism^[[1]](#footnote-1)^**

Given 4 different poems, numbered 1, 2, 3, 4

Denote the criticism type of interest as $T$, and the no-criticism condition as $C$.

Denote $R_{ij}$ as the set of responses^[[2]](#footnote-3)^ that respondents assigned with treatment condition $i \left( i\in\{T,C\} \right)$ for poem $j$ $j\in\{1,2,3,4\}$.

Denote $N_{ij}$ as the number of observations that has treatment condition $i \left( i\in\{T,C\} \right)$ for poem $j (j\in\{1,2,3,4\})$.

Denote $N_{i.}=\sum_{j\in\{1,2,3,4\}} N_{ij}$ as the total number of observations assigned under treatment condition $i \left( i\in T,C \right)$.

***Algorithm.***

| 1 | Initialize $K$, number of replicates required. $K = 300000$ in our analysis. |
| --- | --- |
| 2 | Initialize $V$, an empty vector that stores all the simulated effects (the distribution) |
| 3 | Initialize $w_{Tj}=\frac{N_{Tj}}{N_{T.}}$ for $j\in\{1,2,3,4\}$, $w_{Cj}=\frac{N_{Cj}}{N_{C.}}$ for $j\in\{1,2,3,4\}$ |
|  | for $k$ in 1 to K: |
|  | for $j$ in 1 to 4: |
| 4 | Draw a sample, $R_{Tj}^{*}$, with replacement from $R_{Tj}$ with sample size of $N_{Tj}$ |
| 5 | Draw a sample, $R_{Cj}^{*}$, with replacement from $R_{Cj}$ with sample size of $N_{Cj}$ |
| 6 | Calculate $\mu_{Tj}^{*}$ = mean($R_{Tj}^{*}$), the mean response to criticism $T$ on poem $j$ |
| 7 | Calculate $\mu_{Cj}^{*}$ = mean($R_{Cj}^{*}$), the intrinsic appeal on poem $j$ |
| 8 | Calculate $T^{*}$ = $\sum_{j\in\{1,2,3,4\}} w_{Tj}\mu_{Tj}^{*}$ - $\sum_{j\in\{1,2,3,4\}} w_{Cj}\mu_{Cj}^{*}$, on this replicate |
| 9 | Append $T^{*}$ to $V$ |
| 10 | **return** $V$ |

**Pseudo Code 2: Stratified Bootstrap to Evaluate the Differences of the Effect of a Criticism Between Two Ideology Groups**

Given 4 different poems, numbered 1, 2, 3, 4

Denote the criticism type of interest as $T$, and the no-criticism condition as $C$.

Denote the liberal participants as $L$, and the conservative participants as $R$.

Denote $R_{ijm}$ as the set of responses^[[3]](#footnote-5)^ that respondent group $m$ $\left( m\in\{L,R\} \right)$ assigned with treatment condition $i \left( i\in\{T,C\} \right)$ for poem $j$ ($j\in\{1,2,3,4\}$).

Denote $N_{ijm}$ as the number of responses given by respondent group $m$ $\left( m\in\{L,R\} \right)$ that has treatment condition $i \left( i\in\{T,C\} \right)$ for poem $j (j\in\{1,2,3,4\})$.

Denote $N_{i.m}=\sum_{j\in\{1,2,3,4\}} N_{ijm}$ as the total number of responses given by respondent group $m$, $\left( m\in\{L,R\} \right)$ under treatment condition $i \left( i\in\{T,C\} \right)$.

***Algorithm.***

| 1 | Initialize $K$, number of replicates required. $K = 300000$ in our analysis. |
| --- | --- |
| 2 | Initialize $V$, an empty vector that stores all the simulated differences (the distribution) |
| 3 | Initialize $w_{TjL}=\frac{N_{TjL}}{N_{T.R}}$ for $j\in\{1,2,3,4\}$, $w_{CjL}=\frac{N_{CjL}}{N_{C.L}}$ for $j\in\{1,2,3,4\}$, weights for the poem-specific effects on liberals |
| 4 | Initialize $w_{TjR}=\frac{N_{TjR}}{N_{T.R}}$ for $j\in\{1,2,3,4\}$, $w_{CjR}=\frac{N_{CjR}}{N_{C.R}}$ for $j\in\{1,2,3,4\}$, weights for the poem-specific effects on conservatives |
|  | for $k$ in 1 to K: |
|  | for $j$ in 1 to 4: |
| 5 | Draw a sample, $R_{TjL}^{*}$, with replacement from $R_{TjL}$ with sample size of $N_{TjL}$ |
| 6 | Draw a sample, $R_{CjL}^{*}$, with replacement from $R_{CjL}$ with sample size of $N_{CjL}$ |
| 7 | Calculate $\mu_{TjL}^{*}$ = mean($R_{TjL}^{*}$) , $\mu_{CjL}^{*}$ = mean($R_{CjL}^{*}$), the mean response to criticism $T$ on poem $j$ among liberals as well as the poem’s intrinsic appeal. |
| 8 | Repeat 5-7 to obtain $\mu_{TjR}^{*}$ , and $\mu_{CjR}^{*}$ the mean response to criticism $T$ on poem $j$ among conservatives as well as its intrinsic appeal |
| 9 | Calculate $\mu_{T.L}^{*}$ = $\sum_{j\in\{1,2,3,4\}} w_{TjL}\mu_{TjL}^{*}$, $\mu_{C.L}^{*}$ = $\sum_{j\in\{1,2,3,4\}} w_{CjL}\mu_{CjL}^{*}$ |
| 10 | Calculate $\mu_{T.R}^{*}$ = $\sum_{j\in\{1,2,3,4\}} w_{TjR}\mu_{TjR}^{*}$, $\mu_{C.R}^{*}$ = $\sum_{j\in\{1,2,3,4\}} w_{CjR}\mu_{CjR}^{*}$ |
| 11 | Append $\Delta\mu_{T..}^{*} = (\mu_{T.L}^{*} - \mu_{C.L}^{*}) - (\mu_{T.R}^{*} - \mu_{C.R}^{*})$, the difference of the two simulated effects, to $V$ |
| **Algorithm Cont’d** | |
| 12 | **return** $V$ |

**Pseudo Code 3: Stratified Bootstrap to Evaluate the Differences of the Effect between Two Criticism Types^[[4]](#footnote-6)^**

Given 4 different poems, numbered 1, 2, 3, 4

Denote the first criticism type of interest as $T_{1}$, the second criticism type of interest as $T_{2}$, and the no-criticism condition as $C$.

Denote $R_{ij}$ as the set of responses^[[5]](#footnote-8)^ that respondents assigned with treatment condition $i \left( i\in\{T_{1},T_{2}, C\} \right)$ for poem $j$ $j\in\{1,2,3,4\}$.

Denote $N_{ij}$ as the number of observations that has treatment condition $i \left( i\in\{T_{1},T_{2}, C\} \right)$ for poem $j (j\in\{1,2,3,4\})$.

Denote $N_{i.}=\sum_{j\in\{1,2,3,4\}} N_{ij}$ as the total number of observations assigned under treatment condition $i$ $i \left( i\in\{T_{1},T_{2}, C\} \right)$.

***Algorithm.***

| 1 | Initialize $K$, number of replicates required. $K = 300000$ in our analysis. |
| --- | --- |
| 2 | Initialize $V$, an empty vector that stores all the simulated differences (the distribution) |
| 3 | Initialize $w_{T_{1}j}=\frac{N_{T_{1}j}}{N_{T_{1}.}}$ for $j\in\{1,2,3,4\}$, weight for the poem-specific effects with $T_{1}$ |
| 4 | Initialize $w_{T_{2}j}=\frac{N_{T_{2}j}}{N_{T_{2}}}$ for $j\in\{1,2,3,4\}$, weight for the poem-specific effects with $T_{2}$ |
| 5 | Initialize $w_{Cj}=\frac{N_{Cj}}{N_{C}}$ for $j\in\{1,2,3,4\}$, weight for the poem-specific baseline effects^[[6]](#footnote-9)^ |
|  | for $k$ in 1 to K: |
|  | for $j$ in 1 to 4: |
| 6 | Draw a sample, $R_{T_{1}j}^{*}$, with replacement from $R_{T_{1}j}$ with sample size of $N_{T_{1}L}$ |
| 7 | Draw a sample, $R_{T_{2}j}^{*}$, with replacement from $R_{T_{2}j}$ with sample size of $N_{T_{2}j}$ |
| 8 | Draw a sample, $R_{Cj}^{*}$, with replacement from $R_{Cj}$ with sample size of $N_{Cj}$ |
| **Algorithm Cont’d** | |
| 9 | Calculate $\mu_{T_{1}j}^{*}$ = mean($R_{T_{1}j}^{*}$) , the mean response to criticism $T_{1}$ on poem $j$ |
| 10 | Calculate $\mu_{T_{2}j}^{*}$ = mean($R_{T_{2}j}^{*}$), the mean response to criticism $T_{2}$ on poem $j$ |
| 11 | Calculate $\mu_{Cj}^{*}$ = mean($R_{Cj}^{*}$), the intrinsic appeal on poem $j$ |
| 12 | Calculate $\mu_{T_{1}}^{*}$ = $\sum_{j\in\{1,2,3,4\}} w_{T_{1}j}\mu_{T_{1}j}^{*}$ |
| 13 | Calculate $\mu_{T_{2}}^{*}$ = $\sum_{j\in\{1,2,3,4\}} w_{T_{2}j}\mu_{T_{2}j}^{*}$ |
| 14 | Calculate $\mu_{C}^{*}$ = $\sum_{j\in\{1,2,3,4\}} w_{Cj}\mu_{Cj}^{*}$ |
| 15 | Append $\Delta\mu_{T}^{*} = (\mu_{T_{1}}^{*}- \mu_{C}^{*}) - (\mu_{T_{2}}^{*}- \mu_{C}^{*})$, the difference of the two simulated effects, to $V$ |
| 16 | **return** $V$ |

1. This pseudo code can be applied to responses provided by all respondents or any of the ideology groups [↑](#footnote-ref-1)
2. The responses could be a binary variable indicating whether the respondent agrees with the word associated with the criticism on the specific poem, or the discrete number that corresponds to the Likert-scale questions. [↑](#footnote-ref-3)
3. The responses could be a binary variable indicating whether the respondent agrees with the word associated with the criticism on the specific poem, or the discrete number that corresponds to the Likert-scale questions. [↑](#footnote-ref-5)
4. This pseudo code can be applied to responses provided by all respondents or any of the ideology groups [↑](#footnote-ref-6)
5. The responses could be a binary variable indicating whether the respondent agrees with the word associated with the criticism on the specific poem, or the discrete number that corresponds to the Likert-scale questions. [↑](#footnote-ref-8)
6. In cases where the baseline means differently for different treatments (i.e. agreement to words associated with criticism), the weight is the same since each participant in no-criticism condition will evaluate every poem. [↑](#footnote-ref-9)
