# Supplementary material for: The polarization of literary censorship in the U.S
Source: PLoS One. 2025 Sep 23;20(9):e0332240. doi: 10.1371/journal.pone.0332240 (PMC12456764; doi:10.1371/journal.pone.0332240)
Supplement: S7 File — (DOCX) [file pone.0332240.s009.docx]

**S7 File: Supplement Analysis in Study 2**

**Table A. The proportion of participants who agreed with the words associated with different types of criticism, under the no-criticism condition and decomposed by participant ideology.** Empirical confidence intervals are obtained via bootstrap resampling with 300, 000 replications (see S6 File for the exact bootstrap procedure).

|  | **Intrinsic Appeal** | | | |
| --- | --- | --- | --- | --- |
|  | **Liberals** | **Conservatives** | **Moderates** | **All** |
| Liberal Criticism | 3.2^***^  ($p<0.001)$ | 6.7^***^  ($p<0.001)$ | 3.3^*^  ($p=0.031)$ | 4.0^***^  ($p<0.001)$ |
| Conservative Criticism | 1.4^**^  ($p=0.004)$ | 3.7^**^  ($p=0.004)$ | 1.7  ($p=0.253)$ | 1.9^***^  ($p<0.001)$ |
| Technical Criticism | 12.7^***^  ($p<0.001)$ | 13.4^***^  ($p<0.001)$ | 15.8^***^  ($p<0.001)$ | 13.3^***^  ($p<0.001)$ |
| Positive Criticism | 5.9^***^  ($p<0.001)$ | 6.1^***^  ($p<0.001)$ | 10.0^***^  ($p<0.001)$ | 6.7^***^  ($p<0.001)$ |
| ^***^ *p* < 0.001, ^**^ *p* < 0.01, ^*^ *p* < 0.05, ^+^ *p* < 0.1 | | | | |

**Table B. Proportion of participants who agreed with different types of criticism, net of responses in the no-criticism condition and decomposed by participant ideology.** Empirical confidence intervals are obtained via bootstrap resampling with 300, 000 replications (see S6 File for the exact bootstrap procedure).

|  | **Proportion of participants who agreed with different types of criticism**  **(Net of Intrinsic Appeal)** | | | |
| --- | --- | --- | --- | --- |
|  | **Liberals** | **Conservatives** | **Moderates** | **All** |
| Liberal Criticism | 18.9^***^  ($p<0.001)$ | 13.4^***^  ($p<0.001)$ | 14.5^***^  ($p<0.001)$ | 16.8^***^  ($p<0.001)$ |
| Conservative Criticism | 5.8^***^  ($p<0.001)$ | 7.2^*^  ($p=0.010)$ | 7.3^**^  ($p=0.004)$ | 6.2^***^  ($p<0.001)$ |
| Technical Criticism | 16.6^***^  ($p<0.001)$ | 26.1^***^  ($p<0.001)$ | 15.4^**^  ($p=0.004)$ | 18.6^***^  ($p<0.001)$ |
| Positive Criticism | 10.2^***^  ($p<0.001)$ | 7.9^*^  ($p=0.023)$ | 10.5^*^  ($p=0.021)$ | 9.7^***^  ($p<0.001)$ |
| ^***^ *p* < 0.001, ^**^ *p* < 0.01, ^*^ *p* < 0.05, ^+^ *p* < 0.1 | | | | |

**Table C. Mean favorability score in the no-criticism condition, by participant’s ideological alignment for different ideology groups.** Empirical confidence intervals are obtained via bootstrap resampling with 300000 replications. Favorability score was measured on a 7-point scale where -3 means strongly dislike, 0 means neither like nor dislike, and 3 means strongly like. Empirical confidence intervals, significance, and point estimates are established with 300, 000 replicates using stratified bootstrap. (See S6 File for the exact bootstrap procedure)

|  | **Mean favorability score,**  **Decomposed by ideology group** | | | |
| --- | --- | --- | --- | --- |
|  | **Liberals** | **Conservatives** | **Moderates** | **All** |
| No Criticism | -0.016  ($p=0.841)$ | -0.683^***^  ($p<0.001)$ | -0.308^*^  ($p=0.041)$ | -0.215^***^  ($p<0.001)$ |
| ^***^ *p* < 0.001, ^**^ *p* < 0.01, ^*^ *p* < 0.05, ^+^ *p* < 0.1 | | | | |

**Table D. Mean favorability score under the influence of different types of criticism, by participant’s ideological alignment, net of responses in the no-criticism condition, for different ideology groups.** Empirical confidence intervals are obtained via bootstrap resampling with 300000 replications. Favorability score was measured on a 7-point scale where -3 means strongly dislike, 0 means neither like nor dislike, and 3 means strongly like. Empirical confidence intervals, significance, and point estimates are established with 300, 000 replicates using stratified bootstrap (see S6 File for the exact bootstrap procedure).

|  | **Mean favorability score change (Net of Intrinsic Appeal)**  **Decomposed by ideology group** | | | |
| --- | --- | --- | --- | --- |
|  | **Liberals** | **Conservatives** | **Moderates** | **All** |
| Negative Liberal Criticism | -0.654^***^  ($p<0.001)$ | -0.185  ($p=0.357)$ | -0.290  ($p=0.162)$ | -0.495^***^  ($p<0.001)$ |
| Negative Conservative Criticism | -0.262^*^  ($p=0.025)$ | -0.022  ($p=0.910)$ | 0.022  ($p=0.913)$ | -0.161^+^  ($p=0.082)$ |
| Negative Non-ideological Criticism | -0.450^***^  ($p<0.001)$ | -0.224  ($p=0.249)$ | -0.352  ($p=0.099)$ | -0.383^***^  ($p<0.001)$ |
| Positive Non-ideological Criticism | -0.014  ($p=0.903)$ | 0.280  ($p=0.001)$ | 0.112  ($p=0.592)$ | 0.080  ($p=0.382)$ |
| ^***^ *p* < 0.001, ^**^ *p* < 0.01, ^*^ *p* < 0.05, ^+^ *p* < 0.1 | | | | |

**Table E. Mean agreement with a poem’s publication in absence of the influence of criticism by participant’s ideological alignment, for different ideology groups.** Tendency was measured on a 7-point scale where -3 means strongly disagree with the publication, 0 means neither agree nor disagree, and 3 means strongly agree with the poem’s publication. Empirical confidence intervals and significance are established with 300, 000 replicates using stratified bootstrap (see S6 File for the exact bootstrap procedure).

|  | **Mean agreement with a poem’s publication**  **Decomposed by ideology group** | | | |
| --- | --- | --- | --- | --- |
|  | **Liberals** | **Conservatives** | **Moderates** | **All** |
| No criticism | 0.549^***^  $(p<0.001)$ | -0.134  $(p=0.330)$ | -0.217  $(p=0.151)$ | 0.340***  $(p<0.001)$ |
| ^***^ *p* < 0.001, ^**^ *p* < 0.01, ^*^ *p* < 0.05, ^+^ *p* < 0.1 | | | | |

**Table F. Mean agreement with a poem’s publication under the influence of different types of criticism by participant’s ideological alignment, net of responses in the no-criticism condition.** Confidence intervals and significance were calculated using bootstrap re-sampling with 300000 replications. Tendency was measured on a 7-point scale where -3 means strongly disagree with the publication, 0 means neither agree nor disagree, and 3 means strongly agree with the poem’s publication. Empirical confidence intervals, significance, and point estimates are established with 300, 000 replicates using stratified bootstrap (see Appendix S6 File for the exact bootstrap procedure).

|  | **Change in mean approval of a poem’s publication**  **Decomposed by ideology group** | | | |
| --- | --- | --- | --- | --- |
|  | **Liberals** | **Conservatives** | **Moderates** | **All** |
| Negative Liberal Criticism | -0.528^***^  $(p<0.001)$ | -0.114  $(p=0.580)$ | -0.467^*^  $(p=0.036)$ | -0.436^***^  $(p<0.001)$ |
| Negative Conservative Criticism | -0.410^***^  $(p<0.001)$ | 0.002  $(p=0.991)$ | -0.217  $(p=0.328)$ | -0.298^**^  $(p=0.002)$ |
| Negative Non-ideological Criticism | -0.446^***^  $(p<0.001)$ | -0.176  $(p=0.401)$ | -0.118  $(p=0.607)$ | -0.336^***^  $(p<0.001)$ |
| Positive Non-ideological Criticism | -0.010  $(p=0.931)$ | 0.328  $(p=0.125)$ | 0.248  $(p=0.232)$ | 0.094  $(p=0.308)$ |
| ^***^ *p* < 0.001, ^**^ *p* < 0.01, ^*^ *p* < 0.05, ^+^ *p* < 0.1 | | | | |

**Table G. Mean self-reported comprehension with a poem’s publication in absence of the influence of criticism by participant’s ideological alignment.** Empirical confidence intervals are obtained via bootstrap resampling with 300, 000 replications (see S6 File for the exact bootstrap procedure). Comprehension was measured on a 7-point scale where -3 means extremely hard to understand the poem, 0 means neither hard nor easy, and 3 means extremely easy to understand the poem.

|  | **Mean Self-reported Comprehension Score**  **Decomposed by ideology group** | | | |
| --- | --- | --- | --- | --- |
|  | **Liberals** | **Conservatives** | **Moderates** | **All** |
| No Criticism | 0.396^***^  $(p<0.001)$ | -0.085  $(p=0.518)$ | 0.233  $(p=0.088)$ | 0.250^***^  $(p<0.001)$ |
| ^***^ *p* < 0.001, ^**^ *p* < 0.01, ^*^ *p* < 0.05, ^+^ *p* < 0.1 | | | | |

**Table H. Mean comprehension score under the influence of different types of criticism, by participant’s ideological alignment, net of responses in the no-criticism condition.** Empirical confidence intervals are obtained via bootstrap resampling with 300000 replications (see S6 File for the exact bootstrap procedure). Comprehension was measured on a 7-point scale where -3 means extremely hard to understand the poem, 0 means neither hard nor easy, and 3 means extremely easy to understand the poem.

|  | **Change in mean comprehension score,**  **Decomposed by ideology group** | | | |
| --- | --- | --- | --- | --- |
|  | **Liberals** | **Conservatives** | **Moderates** | **All** |
| Negative Liberal Criticism | -0.218^*^  $(p=0.043)$ | 0.225  $(p=0.256)$ | 0.168  $(p=0.386)$ | -0.041  $(p=0.638)$ |
| Negative Conservative Criticism | -0.233^*^  $(p=0.025)$ | 0.186  $(p=0.359)$ | 0.186  $(p=0.348)$ | -0.058  $(p=0.498)$ |
| Negative Non-ideological Criticism | 0.032  $(p=0.766)$ | 0.310  $(p=0.103)$ | -0.117  $(p=0.584)$ | 0.073  $(p=0.400)$ |
| Positive Non-ideological Criticism | 0.007  $(p=0.950)$ | 0.496^*^  $(p=0.011)$ | 0.088  $(p=0.650)$ | 0.134  $(p=0.110)$ |
| ^***^ *p* < 0.001, ^**^ *p* < 0.01, ^*^ *p* < 0.05, ^+^ *p* < 0.1 | | | | |
