# Supplementary figures and images for: The polarization of literary censorship in the U.S
Source: PLoS One. 2025 Sep 23;20(9):e0332240. doi: 10.1371/journal.pone.0332240 (PMC12456764; doi:10.1371/journal.pone.0332240)

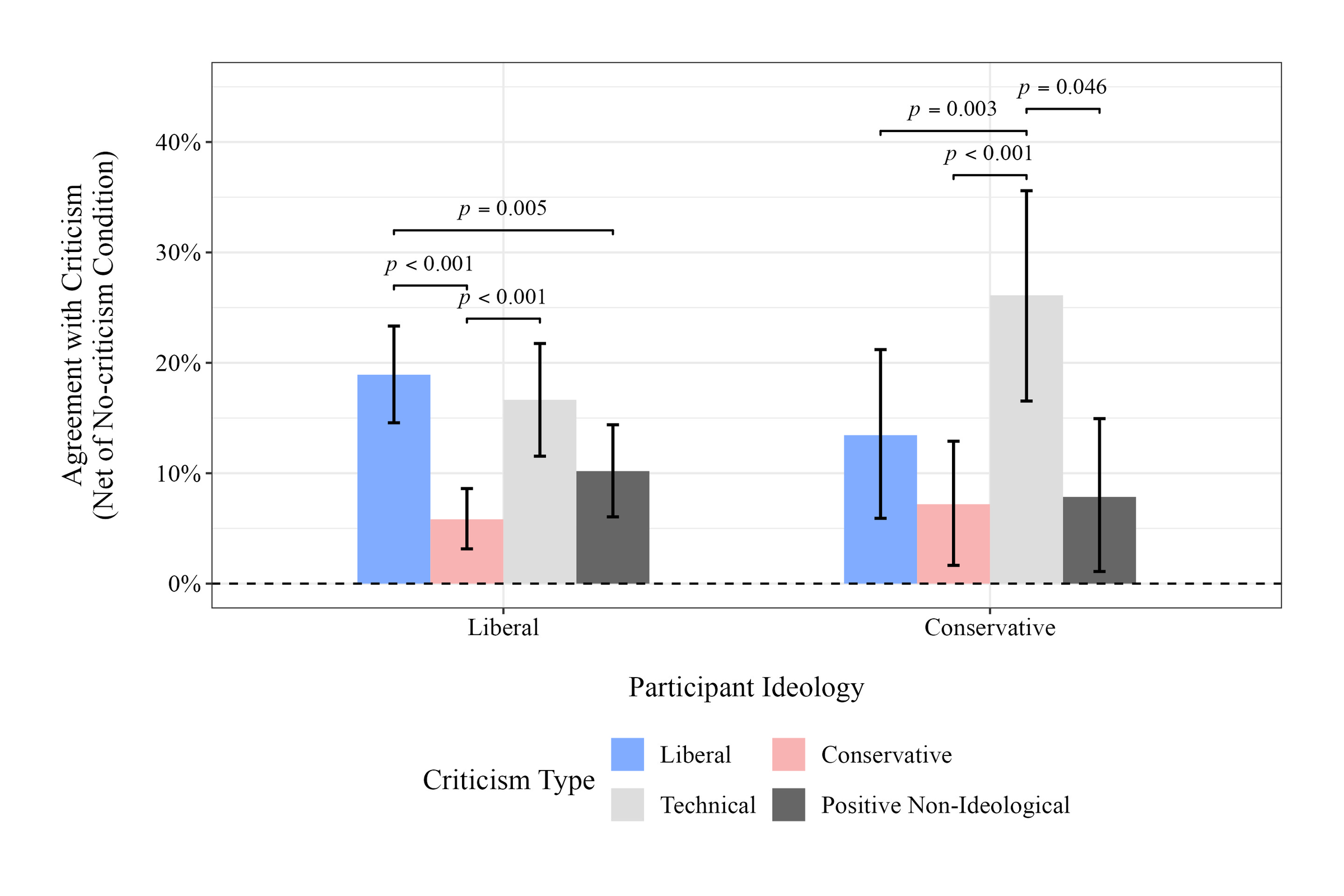

Supplement: S1 Fig — Confidence intervals and significance were calculated using bootstrap re-sampling with 300,000 replications (see S6 File for details). P-values are only displayed for significant differences. (TIF) [file pone.0332240.s010.tif]

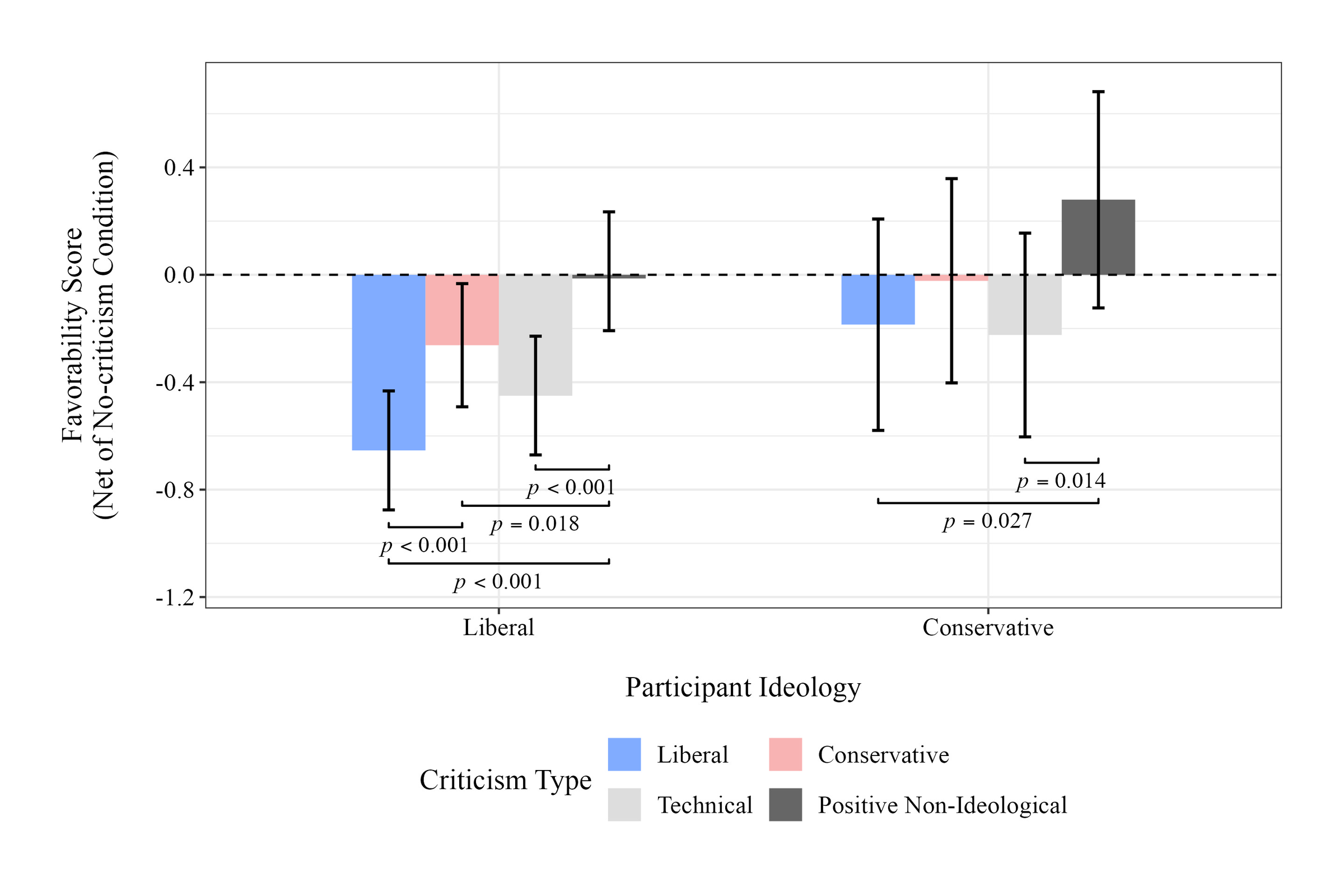

Supplement: S2 Fig — Confidence intervals and significance were calculated using bootstrap re-sampling with 300,000 replications (see S6 File for details). P-values are only displayed for significant differences. (TIF) [file pone.0332240.s011.tif]

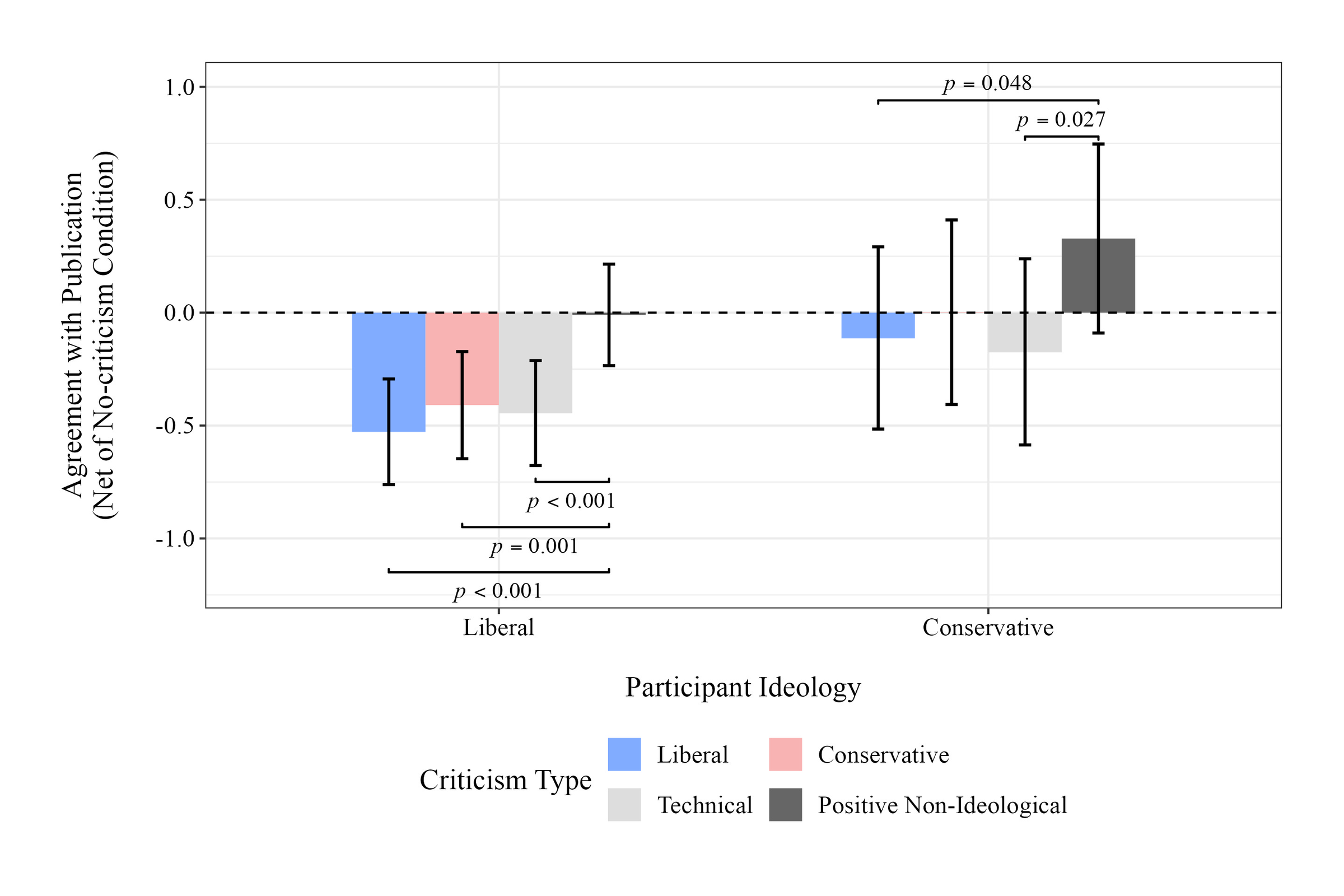

Supplement: S3 Fig — Confidence intervals and significance were calculated using bootstrap re-sampling with 300,000 replications. P-values are only displayed for significant differences. (TIF) [file pone.0332240.s012.tif]

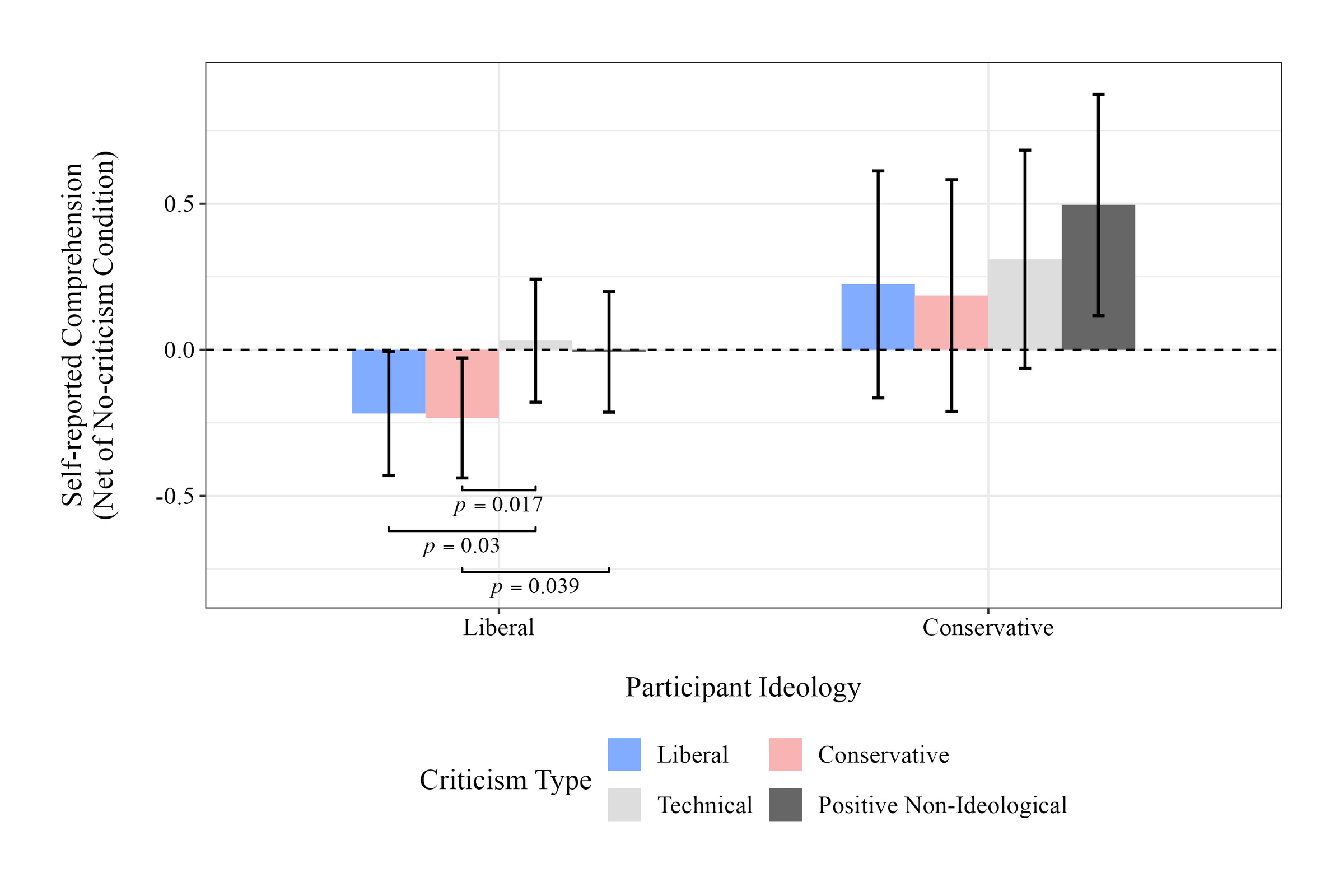

Supplement: S4 Fig — Confidence intervals and significance were calculated using bootstrap re-sampling with 300,000 replications. P-values are only displayed for significant differences. (TIF) [file pone.0332240.s013.tif]
